# Mass campaigns combining antimalarial drugs and anti-infective vaccines as seasonal interventions for malaria control, elimination and prevention of resurgence: a modelling study

**DOI:** 10.1186/s12879-019-4467-4

**Published:** 2019-10-29

**Authors:** Flavia Camponovo, Chris F. Ockenhouse, Cynthia Lee, Melissa A. Penny

**Affiliations:** 1 Swiss Tropical and Public Health Institute, Basel, Switzerland; 2 University of Basel, Basel, Switzerland; 3 PATH’s Malaria Vaccine Initiative, Washington, DC USA

**Keywords:** Vaccine, Mass intervention, Resurgence, Seasonal transmission, Malaria, Simulation

## Abstract

**Background:**

The only licensed malaria vaccine, RTS,S/AS01, has been developed for morbidity-control in young children. The potential impact on transmission of deploying such anti-infective vaccines to wider age ranges, possibly with co-administration of antimalarial treatment, is unknown. Combinations of existing malaria interventions is becoming increasingly important as evidence mounts that progress on reducing malaria incidence is stalling and threatened by resistance.

**Methods:**

Malaria transmission and intervention dynamics were simulated using *OpenMalaria,* an individual-based simulation model of malaria transmission, by considering a seasonal transmission setting and by varying epidemiological and setting parameters such as transmission intensity, case management, intervention types and intervention coverages. Chemopreventive drugs and anti-infective vaccine efficacy profiles were based on previous studies in which model parameters were fitted to clinical trial data. These intervention properties were used to evaluate the potential of seasonal mass applications of preventative anti-infective malaria vaccines, alone or in combination with chemoprevention, to reduce malaria transmission, prevent resurgence, and/or reach transmission interruption.

**Results:**

Deploying a vaccine to all ages on its own is a less effective intervention strategy compared to chemoprevention alone. However, vaccines combined with drugs are likely to achieve dramatic prevalence reductions and in few settings, transmission interruption. The combined mass intervention will result in lower prevalence following the intervention compared to chemoprevention alone and will increase chances of interruption of transmission resulting from a synergistic effect between both interventions. The combination of vaccine and drug increases the time before transmission resurges after mass interventions cease compared to mass treatment alone. Deploying vaccines and drugs together requires fewer rounds of mass intervention and fewer years of intervention to achieve the same public health impact as chemoprevention alone.

**Conclusions:**

Through simulations we identified a previously unidentified value of deploying vaccines with drugs, namely the greatest benefit will be in preventing and delaying transmission resurgence for longer periods than with other human targeted interventions. This is suggesting a potential role for deploying vaccines alongside drugs in transmission foci as part of surveillance-response strategies.

## Background

In 2015 WHO set new goals of reducing malaria incidence by 90% and eliminating malaria in 35 countries by 2030 [1]. This followed dramatic reductions in malaria prevalence from 2000 to 2015 [1] due to scale-up of vector control and artemisinin combination therapy [2]. However, recent estimates indicate that the improvements have stalled, with increasing incidence in several countries [3], and current interventions and implementation strategies may not be sufficient. New tools such as novel insecticides, drugs, and vaccines, are likely to play a role in interruption of malaria transmission [4] and continue to be developed. As we wait for novel tools to be tested and approved, it is important to use existing tools as effectively as possible to help facilitate the efforts of 20 countries in achieving their goal of malaria elimination in the next 7 years as documented by WHO [1]. Increased funding and greater access to interventions, such as effective case management and vector control tools, are essential; it is also increasingly important to innovate the deployment of combinations of existing interventions.

There is just one malaria vaccine that has successfully completed Phase 3 clinical studies, RTS,S/AS01, a pre-erythrocytic or anti-infective vaccine (AIV), targeting sporozoite stages of the *Plasmodium falciparum* life cycle. RTS,S/AS01 received a positive opinion from the European Medicines Agency in 2015 [5], and pilot implementation via routine childhood immunization in regions of Ghana, Kenya and Malawi have started earlier this year [6]. From extensive analysis of Phase 3 trials the vaccine was estimated to have a high initial efficacy, with protection waning over time [7, 8]. Recently published data showed that an altered regimen of RTS,S in which the third dose is delayed and given at a fraction of the original dosage (delayed, fractional dose RTS,S) results in a higher efficacy in human challenge trial [9] and thus may result in a longer duration of protection, although this still needs to be confirmed in the field. There are many vaccines in development targeting sporozoite, liver, blood, or mosquito stages of the malaria parasite, including AIVs such as the delayed fractional dose RTS,S, the whole *Plasmodium falciparum* sporozoite based PfSPZ Vaccine [10, 11], and R21 [12]. A vaccine which can confer many years of protection is desirable but in addition to technical and regulatory challenges there may be biological hurdles that cannot be easily overcome.

Clinical development of RTS,S focused on reducing morbidity and mortality, especially in young children, the rationale being to add additional protection to vector control for the vulnerable population experiencing the highest burden of disease. Modeling studies informed by clinical trial results estimated that, despite waning protection over time, routine immunization of children with RTS,S is likely to have a positive public health impact [13]. Given that duration of protection from RTS,S immunization is shorter than expected, but longer than chemoprevention, alternative uses of the vaccine as a yearly seasonal intervention targeting children is currently being investigated and compared to seasonal malaria chemoprevention (SMC) in clinical studies in Burkina Faso and Mali [14]. Seasonal transmission of malaria due to rainfall patterns is common in the sub-Sahelian region [15], and novel seasonal preventive tools could be of great value in many endemic countries.

To reach and maintain pre-elimination prevalence levels, the entire population of all ages needs to be protected from infection, rather than children only. Mass Drug Administration (MDA) can accelerate prevalence reduction by clearing infections over a short time period, but the chemo-preventative effect is relatively short-lived. MDA is also unlikely to interrupt malaria parasite transmission, since prevalence reduction is transient and likely to be followed by malaria resurgence [16–18]. MDA is currently implemented in Zambia alongside reactive case detection [19], and being considered in the Strategy for Elimination in the Greater Mekong Subregion (2015–2030) to combat drug resistance [20], and to address migrant and mobile populations such as forest workers in regions of Cambodia [21].

An alternative to SMC or MDA with drugs alone is to combine them with vaccines in seasonal settings. To date little has been done to estimate the impact of adding RTS,S or a similar AIV to existing mass treatment strategies. Previous modelling predicted that a long duration of protection is critical for sustained reduction of malaria transmission via mass vaccination [22], so combination with other interventions is expected to be critical for effective mass vaccination with the existing or future AIVs which have limited duration of protection.

There has been no clinical trial combining MDA with an AIV, although drug and vaccine efficacy profiles are known from field trials. Investigating novel applications of existing interventions requires understanding the likely impact over a large number of possible strategies and delivery paths, and it is not feasible to test all combinations in the field. Modelling and simulation lend themselves to providing evidence before moving to demonstration field studies [16]. In this study, we used an existing individual based model of malaria dynamics to understand the role, and potential public health benefits, of an AIV delivered through mass administration alone or in combination with drugs, in a seasonal setting. We estimated the benefits of mass vaccination with respect to elimination, transmission reduction, resurgence, and prevention, for a range of prevalence and health system settings. These modeling outcomes were compared to those produced by modeling use of MDA alone, to estimate the potential for vaccination in addition to treatment to accelerate prevalence reduction or interruption of transmission, and to estimate the effect of mass vaccination on resurgence rates. In order to consider likely next generation AIVs, we considered two AIVs with different protection profiles, a vaccine with equivalent protection profile to RTS,S/AS01 and a vaccine with a longer duration of protection compared to RTS,S/AS01.

## Methods

### Model and setting details

Malaria transmission and intervention dynamics were simulated using *OpenMalaria* [23], an individual-based simulation model of malaria transmission detailed in [24] and [25]. Briefly, *OpenMalaria* combines an individual-based model of malaria in humans [24] with a deterministic model of malaria in mosquitoes [26]. The simulation model includes sub-models of infection of humans [27], blood-stage parasite densities [28], infectiousness to mosquitoes [29], incidence of morbidity and mortality [30, 31]; and immunity is separated in pre-erythrocytic and blood-stage immunity.

A range of transmission levels were investigated by varying the initial entomological inoculation rate (EIR) from 0.5 to 5 which approximates *Pf*PR_2–10_ of 1–20%, depending on the underlying case management (Additional file 1: Figure S10). This is intended to correspond to the residual EIR conditional on vector control measures already in place, which are assumed to remain constant throughout the duration of the simulations. The yearly transmission pattern was chosen as an archetypal highly seasonal setting with a transmission season of 3 months (roughly equating to Senegal where SMC is ongoing [14]). Routine case-management levels were modelled as the 14 day effective treatment coverage (*E*_14_) with a range of 15–80% (typifying both current levels of case management in the vast majority of African countries [32], as well as optimistic scale up of health system improvements). If not otherwise specified, results in the main analysis correspond to E_14_ = 45%. Three levels of imported infections were investigated when analyzing resurgence (0, 2 and 20 infections per 1000 population). A population of 10,000 individuals was assumed, and 10 stochastic realizations of the model were simulated for each strategy and setting combination (total of 847,000 simulations). Initial parasite prevalence levels in 2–10 year olds (*Pf*PR_2–10_), levels of effective access to care (E_14_), and timing and coverage levels of the interventions are summarized in Additional file 1: Tables S1 and S2.

### Intervention properties

**Fig. 1:**
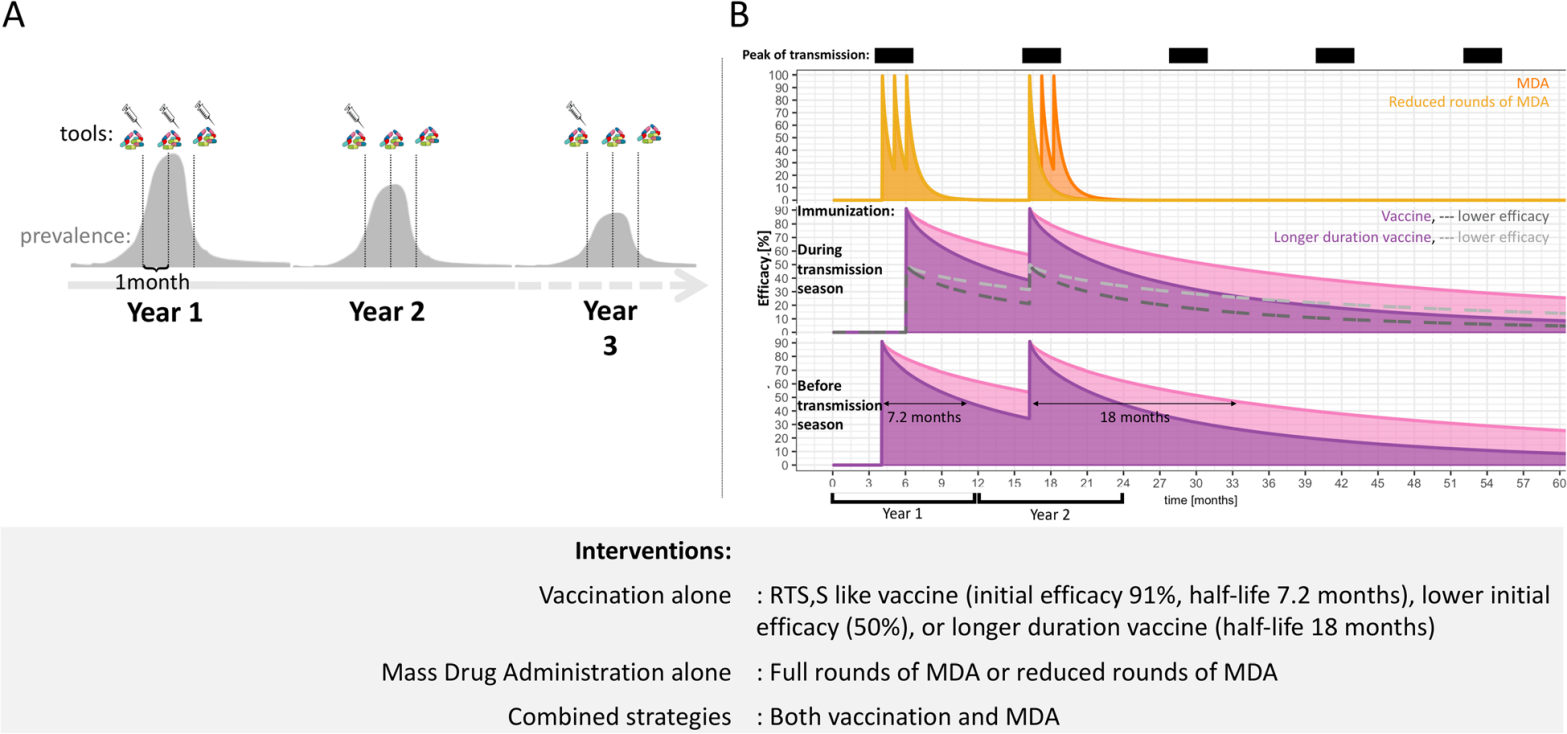
Main simulated strategies and drug and vaccine efficacy profiles. **a** Illustrated strategies: MDA alone, vaccine alone, or MDA with vaccine. MDA application alone is 3 rounds coinciding with the pattern of seasonal transmission, with 2–3 years of 3 rounds or 3 rounds for only the first year followed by 1–2 years of 1 round at the beginning of the transmission season; RTS,S-like-duration vaccine or longer duration vaccine application alone is 3 rounds coinciding with the pattern of seasonal transmission with 1–2 years of 1 dose at the beginning of the season or as 3 rounds before the pattern of seasonal transmission, with 1–2 years of 1 dose at the beginning of the season; and strategies combining MDA with RTS,S-like-duration vaccine or longer duration vaccine are a combination of all MDA and vaccine implementations combined together. **b** Efficacy against infection profiles of 2 years of intervention with MDA, RTS,S like vaccine, vaccine with longer duration of protection, and vaccine with lower initial efficacy. x-axis represents the time in months, and efficacy against infection is indicated on the y-axis for MDA given at 1 months interval during 3 months the first year and 1 (yellow) or 3 (orange) rounds the second year, and for mass vaccination with a RTS,S like vaccine (purple), a longer duration protection vaccine (pink), or vaccines with lower initial efficacy against infection (dashed lines). Mass vaccination is delivered either before the peak of transmission the first year (bottom plot) or during the transmission season (middle plot). The first year 3 doses of vaccination are administered, but only the 3rd dose of vaccination is modeled, and the second year only one dose is administered. Initial efficacy against infection for both vaccines are 91% or 50% and the half-life, which is the time in which protection against infection has decayed to half the value of the initial level, is 7.3 months for the RTS,S like vaccine and 18 months for the vaccine with extended duration of protection, indicated with arrows on the diagram. Period of peak transmission are indicated by black boxes

Intervention properties for both vaccination and MDA, are summarized in the Additional file 1: Table S1, and Fig. 1. The underlying parameterization of the vaccine was informed by previous model fitting to RTS,S/AS01 Phase 3 trial results [13], and should be considered to encompass vaccine parameterizations of other AIV in development. The clinical trials reported an observed efficacy against clinical cases in children receiving four doses (5–17 months of age at first dose) of 43.9% (95% CI 39.7–47.8%) over 32 months of follow-up, and with a decline over time [33], which was the basis for fitting the vaccine efficacy for our microsimulations [13]. The action of an AIV in our microsimulation is to prevent infections, which has a consequential effect on preventing clinical disease. The underlying vaccine efficacy against infection in time (including likely decay of effect), defined as the proportionate reduction in force of infection assuming beta distributed variation in efficacy, was parameterized via fitting of our microsimulation to extensive Phase 3 clinical trial data of RTS,S/AS01 and has been described elsewhere [7]. Briefly, AIVs are modelled in *Openmalaria* as preventing new infections via stochastic process with the probability of preventing an infection referred to as the efficacy against infection (also the proportion of blood-stage infections averted). The time course of efficacy was described as a weibull function capturing possible exponential, biphasic and step-like decays. This decay function is described via the following equation:

$$ \upvarepsilon ={\upvarepsilon}_0\exp \left(\frac{-{\left(\mathit{\log}2\right)}^{\frac{1}{k}}t}{L^k}\right), $$

where ε_0_ is the initial value of efficacy against infection, *L* is the half-life of decay (time till efficacy against infection is half of the initial value), and parameter *k* informs the shape of the decay (*k* = 1 is exponential).

Bayesian MCMC was used to compare simulated incidence and Phase 3 trial incidence in order to find the most appropriate vaccine properties in *OpenMalaria* defined as efficacy against infection. Models were simultaneously fitted to both control and vaccinated incidence from the trial, and estimates of vaccine properties, site-specific access to care, and the extent of within-site variability were obtained.

This model fitting resulted in an estimated efficacy against infection to be high following immunization, 91% [8], and this efficacy wanes quickly over time resulting in limited duration of protection with the half-life against infection, *L*, of less than 1 year (efficacy of 45.5% at 7.3 months, and *k* = 0.69 describing the shape of efficacy decay) [7, 8]. This half-life refers to time in which protection against infection has decayed to half the value of the initial level. We note decay of this protection is biphasic so that protection continues after this time and decays more slowly than the first 7 months (Fig. 1). This efficacy parametrization is in alignment with the estimates of other modelling groups [13]. In addition to this vaccine parametrization, we have investigated a vaccine with longer duration of protection with a half-life of 1.5 years, and in a subset of settings a vaccine with lower efficacy, reducing initial efficacy against infection from 91 to 50% (Additional file 1: Table S1, and Fig. 1). The fourth dose of the vaccine, and the fifth dose in the 3 years deployment strategies, is assumed to have the same efficacy profile and coverage as the third dose.

Strategies including MDA were implemented in the model with Dihydroartemisinin piperaquine (DHAp), which has slightly lower treatment failure rates than arthemeter-lumefantrine [34] and was the main mass drug used in southern Zambia field studies assessing the short term impact of MDA [35].

### Deployment strategies

The strategies simulated were 1) deployment of mass vaccination of an initial 3 doses in the first year delivered to population older than 6 months of age at first dose (minimum 9 months old at 3rd dose) with 3 monthly spaced doses and yearly single dose vaccination following first year for 2 or 3 years; 2) deployment of MDA of up to 3 monthly rounds per year delivered to ages from 6 months old for 2 or 3 years; or 3) the combination of both interventions (Additional file 1: Table S2). MDA was assumed deployed at the beginning of the transmission season, with an optimistic strategy of 2 additional rounds deployed at 1 month intervals during the transmission season. Either all 3 MDA rounds were implemented every year of the intervention up to 3 years, or only the first round was kept for the following year(s). The vaccine was delivered either prior (with the 3rd dose completed at the beginning of the season) or during the transmission season, the latter possibly favored for logistical reasons as a fewer number of intervention rounds in the combination strategies are required assuming drug and vaccination are delivered at the same time. Vaccination coverage represents the proportion of population receiving all three doses, with no efficacy assumed if receiving less than 3 doses in the first year. The proportion of the population receiving a drug or vaccine (full 3 dose regimen) was randomly allocated for each round and intervention given coverage in the default setting, and in a subset of simulations, the vaccine and MDA, when delivered simultaneously, was administered to the same population for a given coverage. A range of coverages from 40 to 100% were considered.

### Outcome measured as prevalence reduction

We estimated the relative reduction in prevalence achieved using different intervention deployment as the prevalence reduction predicted to be reached each year in a given setting, namely ${D}_t=100\left(1-\frac{P_s(t)}{P_c(t)}\right)$, where *D*_t_ is the relative reduction of the mean prevalence at year *t* of strategy *s*; *P*_*s*_(*t*) the prevalence at time *t* of strategy *s* and *P*_*c*_(*t*) the prevalence at time *t* of the control strategy. The relative maximum prevalence reduction, directly following the intervention deployment, is simply max(*D*_*t*_).

### Outcome measured as interruption of transmission and estimated synergistic behavior of the combined intervention

To estimate the chances of an intervention strategy to achieve elimination in our model we calculated the proportion of simulations achieving interruption of transmission over all stochastic realizations for each setting. The synergistic behavior of MDA combined with mass vaccination in chances to reach elimination was evaluated. The combined mass vaccination with MDA interventions were considered synergistic if the chance to interrupt transmission was greater than the sum of the separate interventions and was defined consistent with previous disease intervention analyses [36]. The level of synergy for differing coverages and transmission settings was calculated as $\upsigma =\left(\frac{\Phi_{\mathrm{combined}}-{\Phi}_{\mathrm{control}}}{\left({\Phi}_{\mathrm{MDA}}-{\Phi}_{\mathrm{control}}\right)+\left({\Phi}_{\mathrm{vaccine}}-{\Phi}_{\mathrm{control}}\right)}\right)-1$, where *ϕ* represents the probability of interrupting transmission in a simulation set denoted in the subscript, namely with no intervention (*control*), MDA alone (*MDA*), mass vaccination alone (*vaccine*), or the combined MDA and vaccination (*combined*). Values of *σ* greater than 0 represent synergism so that the estimated effect of the two combined interventions is greater than additive, values of 0 imply the combined interventions are not more than additive, and values less than 0 imply less than additive or maximum level was reached by one or both single interventions.

### Outcome measured as the rate of resurgence

Where no elimination occurred, we estimated the time it takes after the mass interventions cease for prevalence to return to initial levels, by estimating resurgence rates. The rate of resurgence was assumed to follow a sigmoidal curve and was defined as ${\mathrm{R}}_{\mathrm{t}}=\frac{{\mathrm{P}}_{\mathrm{c}}\left(\mathrm{t}\right)-{\mathrm{P}}_{\mathrm{s}}\left(\mathrm{t}\right)}{{\mathrm{P}}_{\mathrm{c}}\left(\mathrm{t}\right)-{\mathrm{P}}_{\mathrm{min}}}\propto {\left(1+\frac{x^b}{\uplambda_{50}\ }\right)}^{-1}$, where P_min_ is the minimum prevalence reached by strategy *s*, λ_50_ is the estimated half-life of resurgence or time till 50% resurgence representing the years after maximum prevalence reduction was reached in which prevalence resurges by 50% of the reduction, and *b* is the Hill’s slope, representing the steepness of the logistic curve. Similarly, we estimated λ_10_ is the time till 10% resurgence representing the years after maximum prevalence reduction was reached in which prevalence resurges by 10% of the reduction. Resurgence parameters were estimated for strategies with 60% coverage for 2 years of deployment.

## Results

### Impact on time course of prevalence

**Fig. 2:**
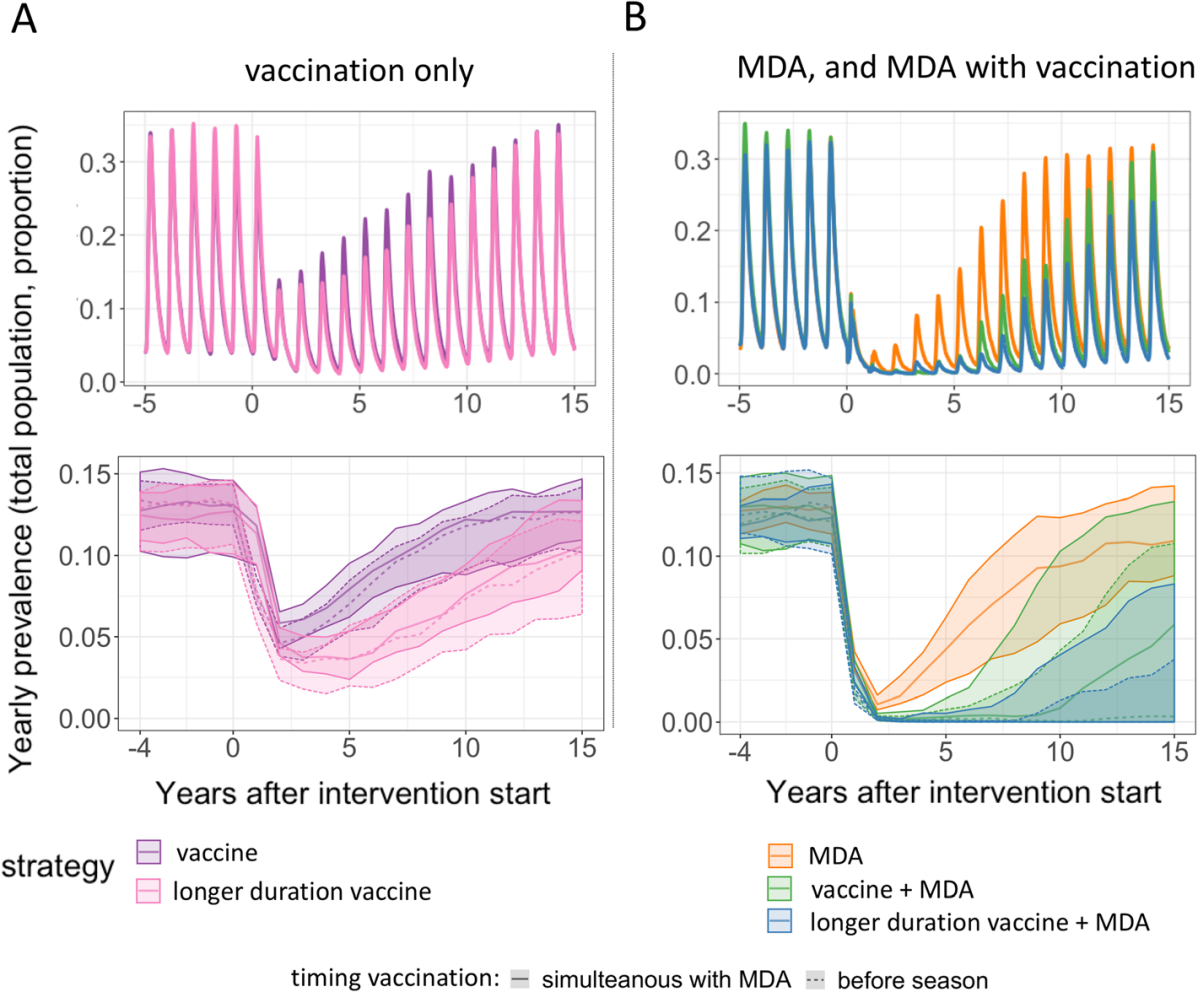
Example time course of predicted all age prevalence following intervention. Single simulation examples (chosen at random) of estimated continuous (upper plots) and median and range of estimated yearly average (lower plots) all age prevalence following different intervention strategies deployed during 2 years. **a** estimated all age prevalence following mass vaccination (purple) or mass vaccination with longer duration vaccine (pink), **b** estimated all age prevalence following full rounds of MDA alone (orange) or in combination with mass vaccination (green) or mass vaccination with longer duration vaccine (blue). Intervention coverage of 60% was assumed, with an initial yearly average *Pf*PR_2–10_ = 4% with peak *Pf*PR_2–10_ ≈ 10–15% (corresponding to an initial EIR of 2 and effective access to care E_14_ = 45%). Full description of the different strategies can be found in Additional file 1: Table S2

Use of a vaccine with equivalent protection profile to RTS,S/AS01 (with high initial efficacy, waning over time reaching half of initial efficacy after 7.3 months) fitted to the Phase 3 clinical trial data [33], or an AIV with longer duration of protection (same initial efficacy but with extended duration of protection, reaching half of initial efficacy after 18 months), or an AIV with lower initial efficacy against infection of 50%, was compared to MDA as single mass interventions alone or in combination, over 2 to 3 years of intervention, as schematized in Fig. 1a, with efficacy profiles illustrated in Fig. 1b. Example time courses of predicted all age prevalence before and after 2 years of mass intervention are illustrated for one simulation from each strategy and across all simulations as average yearly prevalence (Fig. 2, variability shown by minimum and maximum range) (additional strategies shown in Additional file 1: Figures S1–S2). The difference between intervention strategies is most distinguishable at the annual peak of prevalence. MDA and/or mass vaccination strategies are found to effect prevalence in one of two ways, either the strategy successfully interrupts transmission, or the strategy leads to a rapid drop in prevalence followed by malaria resurgence back to initial prevalence levels.

### Relative prevalence reduction

**Fig. 3:**
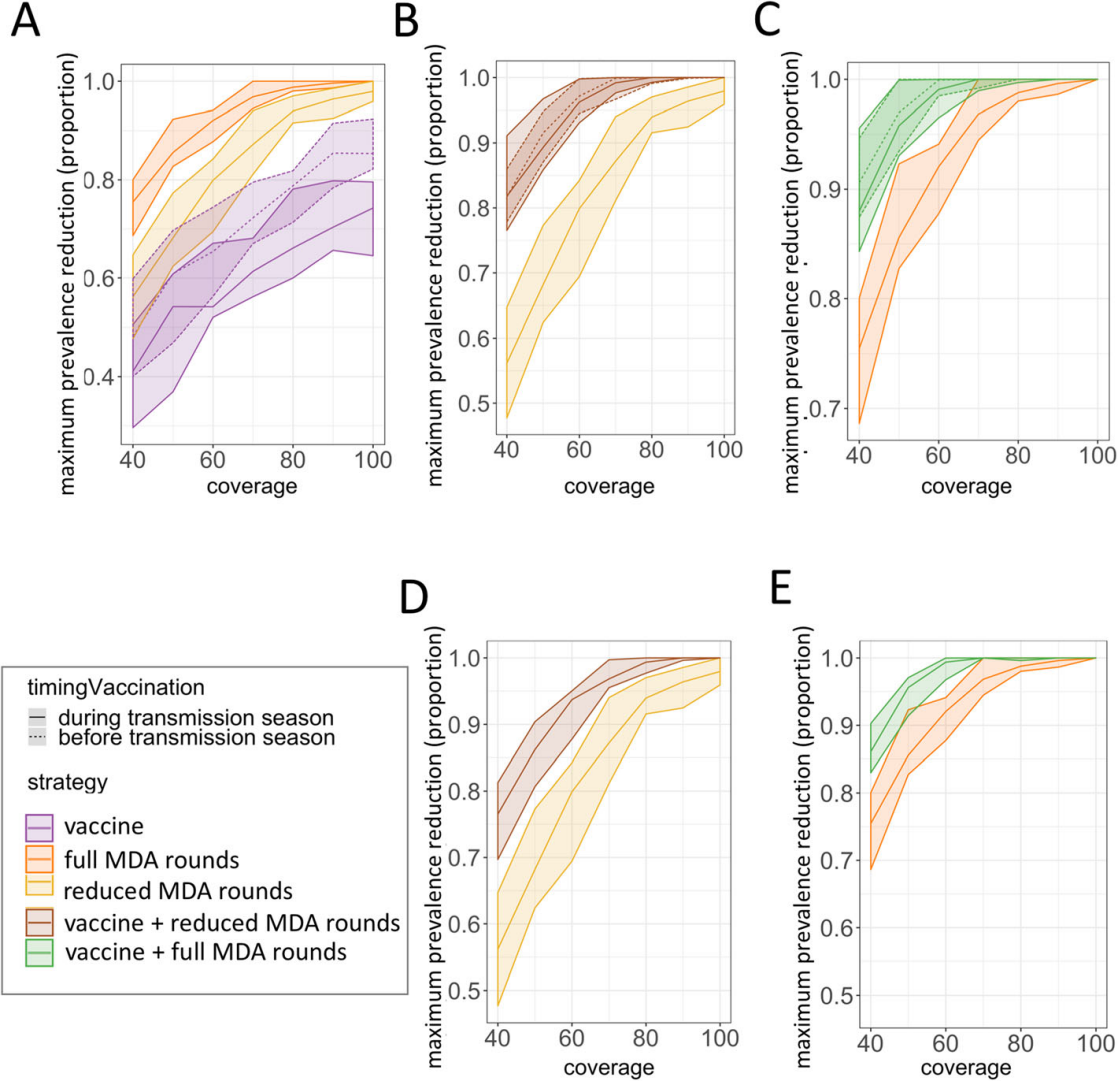
Relative maximum prevalence reduction achieved immediately following 2 years of intervention for different intervention coverages. Estimated maximum prevalence reduction achieved (proportion, where 0 indicates no prevalence reduction and 1 interruption of transmission) for coverage levels 40–100% (x-axis) by **a** single interventions, **b**-**d** combined interventions with drugs and vaccination given independently, and **d**-**e** combined interventions with drugs and vaccination given to the same population when administered simultaneously. **a** Compares MDA or mass vaccination alone: mass vaccination (purple) before (dashed lines) or during (solid lines) transmission season and full rounds of MDA (orange) or reduced rounds of MDA (yellow); **b** and **d** Compare reduced rounds of MDA (yellow) with combined strategies of mass vaccination before (dashed lines) or during (solid lines) the transmission season together with full rounds of MDA (brown); and **c** and **e** Compare reduced rounds of MDA (orange) with combined strategies of mass vaccination before (dashed lines) or during (solid lines) the transmission season together with full rounds of MDA (green). Each intervention is represented by the median and minimum-maximum range across 10 simulations per a strategy. Initial average *Pf*PR_2–10_ = 4% with peak *Pf*PR_2–10_ ≈ 10–15% (corresponding to an initial EIR = 2 and effective access to care E_14_ = 45%)

The impact of different intervention strategies was estimated by comparing the relative reduction in prevalence achieved each year, defined as the yearly prevalence reduction measured for each intervention strategy relative to the prevalence in the control strategy (i.e. no intervention deployed). MDA alone was predicted to be more effective than mass vaccination alone in reducing prevalence, and thus also avoiding resurgence, (Fig. 3a), in settings with different initial prevalence levels (EIR) and case management levels (E_14_) (Additional file 1: Figure S4). Vaccination only strategies in which three doses of vaccine were given prior to seasonal transmission in the first year resulted in larger reductions in prevalence compared to when delivered during the first season (Fig. 3a and Additional file 1: Figure S4). For combined intervention strategies of mass vaccination added to MDA, the predicted relative maximum prevalence reduction achieved was higher compared to strategies deploying MDA alone (Fig. 3b-e), with a predicted higher prevalence reduction when the drug and vaccine was delivered independently from each other (random coverage, Fig. 3b-c) compared to when given simultaneously to the same people (same coverage, Fig. 3d-e). Delivering the 3 doses of vaccine prior to seasonal transmission the first year instead of during the seasonal transmission had little value (Fig. 3b-c)). MDA alone achieved large reductions in prevalence, especially at higher coverage levels and low transmission levels, such that adding mass vaccination had only a limited relative impact on prevalence reduction at these higher coverage levels (Additional file 1: Figure S5).

### Interruption of transmission

**Fig. 4:**
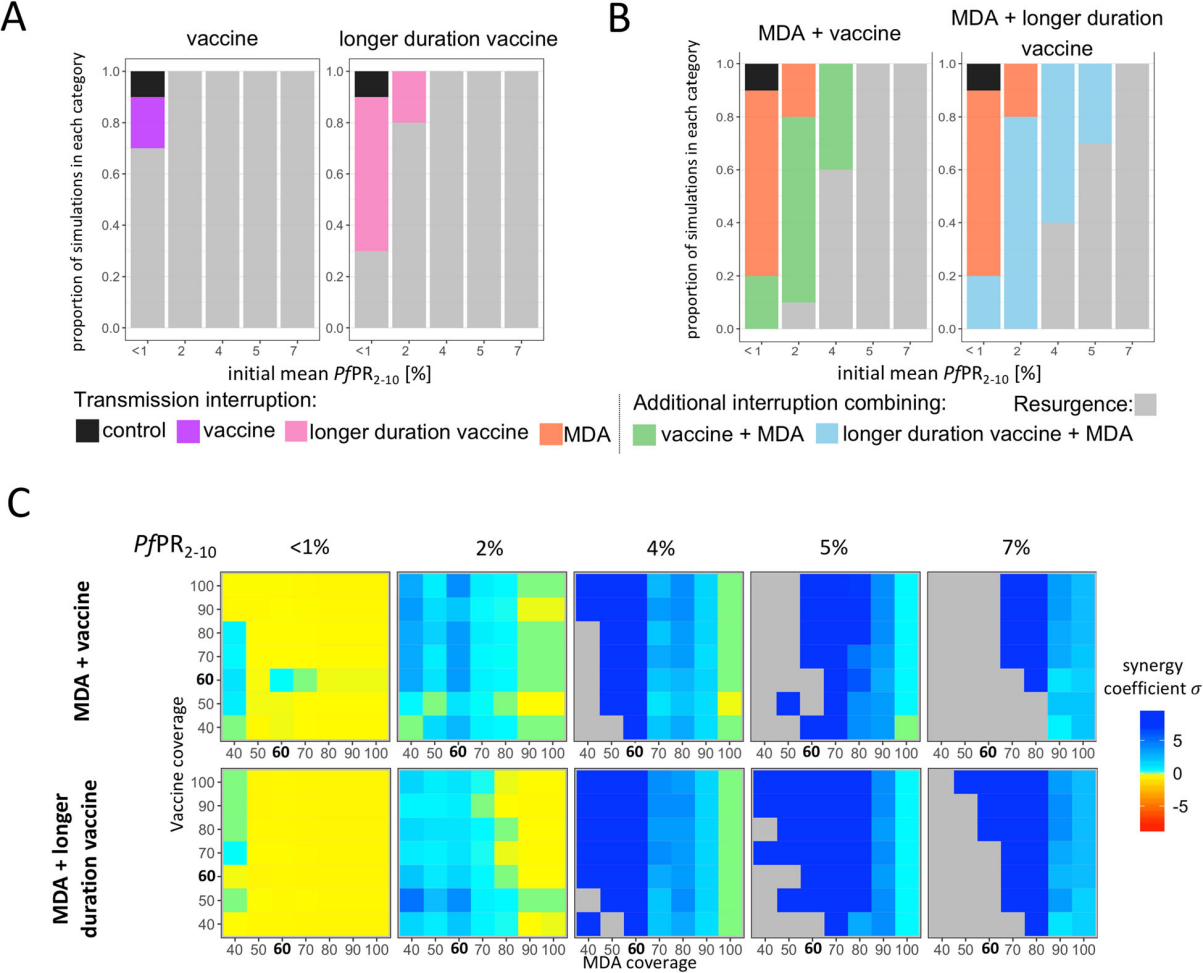
Interruption of transmission and synergism for different intervention strategies. **a**-**b** Proportion of simulations in which interruption of transmission is estimated to be achieved with mass vaccination (**a**) or MDA combined with mass vaccination compared to MDA only (**b**). Initial *Pf*PR_2–10_ (%) levels are shown on the x-axis, and proportion of the simulations falling into each category are shown on the y-axis. All interventions were deployed for 2 years at a coverage of 60%. Categories of simulations are i) interruption of transmission occurred with no intervention at all, due to very low initial prevalence (black), ii) interruption of transmission occurred with single interventions, namely with mass vaccination with RTS,S like vaccine (purple) or longer duration vaccine (pink), or with MDA (orange), iii) interruption of transmission occurred only adding mass vaccination to MDA (green and blue using with RTS,S like vaccine or longer duration vaccine respectively), and iv) resurgence occurred and no interruption of transmission was achieved (grey).**c** Estimated synergy coefficient (σ) of the combined mass vaccination and MDA intervention in regards probability to interrupt transmission. The x-axis indicates coverage levels of MDA, and the y-axis coverage levels of mass vaccination, and the level of synergy between the two intervention strategies are indicated in colour. Blue represents synergistic behavior (> 0) in the combined MDA and mass vaccination, light green represents values of 0 which imply the combined interventions are not more than additive, and yellow to red colours represent values less than 0 which imply less than additive or maximum level was reached by one or both single interventions. Grey areas represent settings where resurgence occurred in all simulation, thus no synergy could be calculated. The synergy coefficient are shown for the combination of MDA with RTS,S like vaccine (upper row) and MDA with the longer duration vaccine (bottom row), and for different levels of initial *Pf*PR_2–10_ (%) (columns)

The proportion of simulations achieving interruption of transmission for each strategy was calculated over all stochastic realizations for each setting. Assuming 60% coverage and 2 years of intervention, interruption of transmission occurred in 10% of the control simulations with initial *Pf*PR_2–10_ less than 1%, highlighting possible stochastic extinction in the models at very low prevalence [17]. Interruption of transmission for all strategies was dependent on initial *Pf*PR_2–10_ with proportion of simulations in which transmission was interrupted decreasing with increasing initial *Pf*PR_2–10_ (Fig. 4a-b), and thus interruption of transmission was more likely in a setting where vector control is already at high coverage (expressed in this study as low initial prevalence). Interruption of transmission was less likely to occur with lower case management (Additional file 1: Table S5 and Figure S6), with MDA, mass vaccination or combined strategies more likely to interrupt transmission if effective access to care is higher. Excluding simulations with initial *Pf*PR_2–10_ under 1% where stochastic extinction can occur, mass vaccination alone did not lead to transmission interruption when using RTS,S-like-duration vaccine and led to transmission interruption in 20% of the simulations in settings of initial *Pf*PR_2–10_ of 2% when using the vaccine with longer duration of protection (Fig. 4a). With a vaccine of lower vaccine efficacy (50%), no interruption of transmission for *Pf*PR_2–10_ higher than 1% was observed. Similarly, at initial *Pf*PR_2–10_ of 2% the proportion of simulations with transmission interruption was 20% for strategies deploying MDA alone. For initial *Pf*PR_2–10_ greater than 4%, transmission interruption was less likely for both vaccination alone and MDA alone (Fig. 4a-b). Combining both interventions at 60% coverage, the highest proportion of simulations in which interruption of transmission was achieved for 2 years of intervention was estimated for initial *Pf*PR_2–10_ range 2–4%, with interruption of transmission estimated at 75% for strategies deploying MDA combined with immunization with a vaccine with equivalent protection properties to RTS,S/AS01, and of 80% when combined with the vaccine with longer duration of protection (Fig. 4b). Including a 3rd year of intervention in these settings increased the proportion to 95–100%, even assuming a coverage of 60% (Additional file 1: Table S3). Combining MDA with a vaccine with lower efficacy resulted in lower chances to interrupt transmission compared to combined strategies with a vaccine with higher efficacy profile, however, for a *Pf*PR_2–10_ of 2%, this lower efficacy vaccine when combined with MDA increased the chances of interrupting transmission from 20 to 60% and from 20 to 70% with longer duration vaccine compared with using MDA only (Additional file 1: Figure S7). Administering the vaccine and MDA simultaneously to the same population for a given coverage level resulted in slightly lower predictions of chances to interrupt transmission than with random coverage between the two interventions (Additional file 1: Figure S6). Proportions of simulations with transmission interruption were similar for strategies vaccinating the population before the transmission season or during the transmission for mass vaccination or combined interventions, even if slightly higher when vaccinating before the season (Additional file 1: Table S3). Further results can be found in Additional file 1: Tables S3-S5.

Synergism, defined as observing a greater proportion of simulations with interrupted transmission in the combined interventions than in the sum of the single interventions (see *methods*), was calculated for a range of initial *Pf*PR_2–10_ and intervention coverage levels (Fig. 4c and Additional file 1: Figure S7 and Figure S9). The lower the initial *Pf*PR_2–10_, the less likely synergism was observed, as the chances to interrupt transmission using MDA as a single intervention was higher. Similarly, increasing case management or increasing MDA coverage decreased synergism between the two interventions. Increasing vaccine coverage or duration of protection increased the chances to interrupt transmission but did not have an obvious effect on synergism. The lower initial vaccine efficacy decreased the number of settings where synergism was predicted (Additional file 1: Figure S7). Synergism was predicted for both scenarios of when the interventions were delivered independently from each other or simultaneously to the same target population (Fig. 4c and Additional file 1: Figure S7).

### Resurgence

**Fig. 5:**
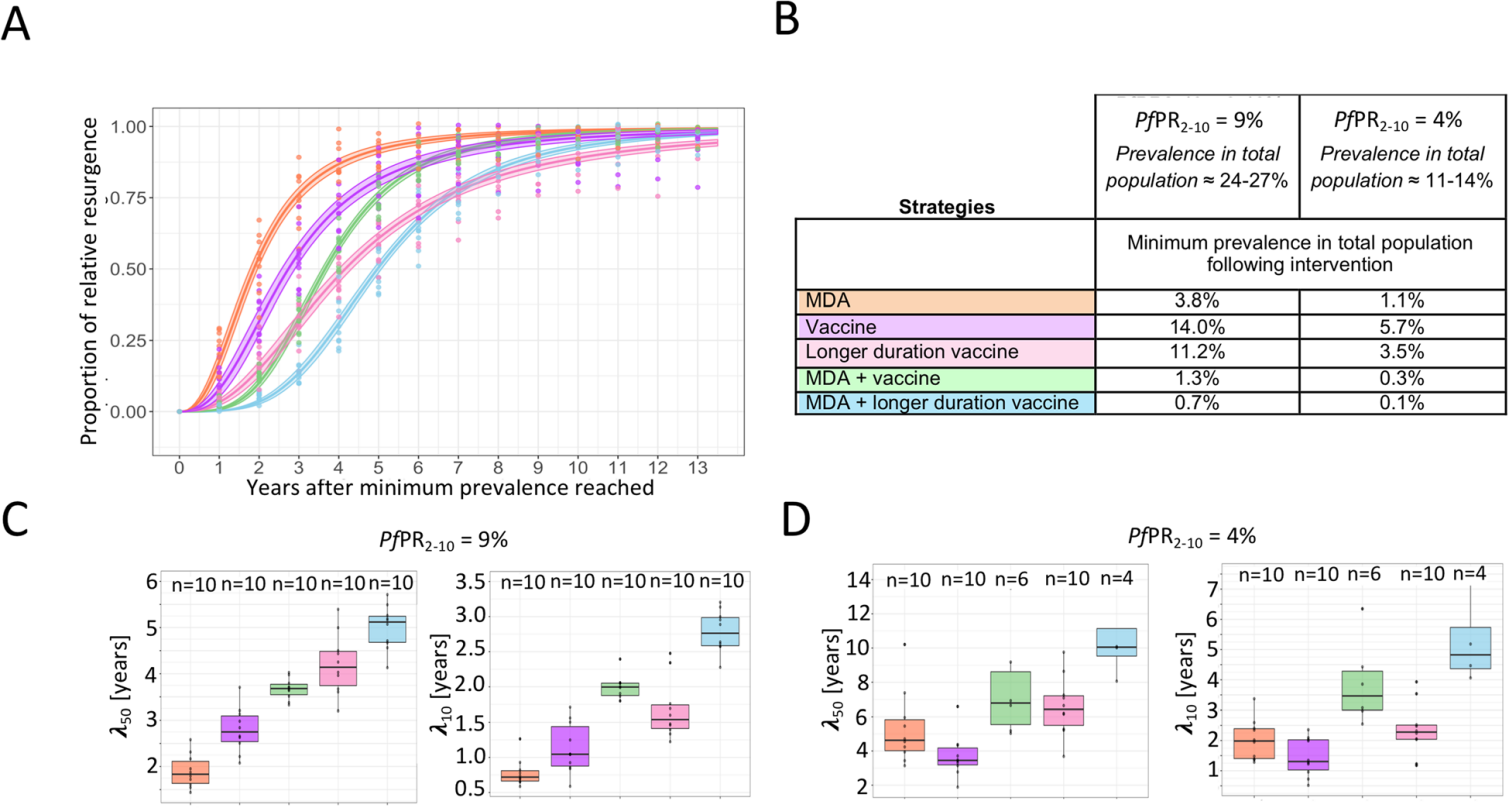
Resurgence following 2 years of deployment of different interventions at *Pf*PR_2–10_ = 4 and 9%. **a** Predicted relative resurgence at initial *Pf*PR_2–10_ = 9% (EIR = 2 and E_14_ = 45%) with 95% confidence intervals estimated from fitting a 4-parameter logistic regression to the pooled simulations for strategy MDA alone (orange), mass vaccination alone (purple), mass vaccination with a longer duration vaccine (pink), MDA combined with mass vaccination (green) and MDA combined with a longer duration vaccine (blue). **b** The average minimum prevalence in the total population reached directly following each intervention. **c**-**d** Summarized parameters describing resurgence (boxplots with median and 95% confidence intervals) estimated from the logistic regressions to each simulations for each strategy are the estimated half-life or time till 50% resurgence, λ_50_, representing the years after maximum prevalence reduction was reached in which prevalence resurges by 50% of the reduction and the time till 10% resurgence, λ_10_, representing the years after maximum prevalence reduction was reached where prevalence resurges 10% of the reduction. The estimates are shown for initial *Pf*PR_2–10_ = 9% in **c** and for initial *Pf*PR_2–10_ = 4% in **d**. The corresponding number of simulations with resurgence per strategy and setting are indicated at the top of each summary boxplot

Resurgence occurs in each simulation in which transmission was not interrupted with prevalence eventually returning to initial levels. Additional to predicting the chances to achieve transmission interruption (or risk of resurgence), we estimated the time to resurgence after deployment of the intervention cease. This time reflects a period where prevalence remains at very low levels and other interventions such as reactive case detection (not simulated here) could potentially be effective to interrupt transmission. In settings of initial *Pf*PR_2–10_ = 9% resurgence occurred in all simulations, and the estimated resurgence half-life, λ_50,_ was lowest for the MDA strategy (median λ_50_ = 1.83 years), whereas for the strategy combining MDA with mass vaccination with RTS,S-like-duration vaccine λ_50_ was more than 2.5 years longer than MDA alone (median λ_50_ = 3.68 years) (Fig. 5 and Additional file 1: Table S6). The estimated Hill’s slope (median 3.55) was also higher, as was λ_10_ (increased by 1.28 years), defined as the time after maximum prevalence reduction was reached in which prevalence resurges to 10% of the reduction, indicating that resurgence was delayed in the combined strategy and prevalence remained lower for a longer time period of time compared to MDA alone. Half-life of resurgence were similar in the strategies where mass vaccination was performed before the transmission season, with no evident increase (Additional file 1: Table S6). For strategies with MDA combined with mass vaccination using a vaccine with equivalent protection profiles to RTS,S/AS01, the half-life of resurgence was increased by 1.28 years if the interventions were deployed for 3 years instead of 2 years.

For initial *Pf*PR_2–10_ of 4%, resurgence parameters were calculated for 80% of the simulations in which resurgence occurred. In the strategy combining MDA with mass vaccination using a vaccine with equivalent protection profile to RTS,S/AS01, λ_50_ was estimated to be more than 2 years longer than MDA alone (median λ_50_ = 6.78 years compared to median λ_50_ = 4.63 years) (Fig. 5 and Additional file 1: Table S6), and the time after maximum prevalence reduction was reached in which prevalence resurges to 10% of the reduction increased by 1.48 years (median λ_10_ = 3.46 against median λ_10_ = 1.98 for MDA only).

Overall, delivering the vaccine to the same people as receiving MDA produced similar estimates of resurgence rates for both prevalence levels investigated (Additional file 1: Table S6). Using a vaccine with lower initial efficacy in the combined strategies had less impact in delaying resurgence but nevertheless the resurgence half-life was estimated to be increased by almost a year compared to using MDA only (Additional file 1: Table S6). Simulations that assumed increased levels of imported infections resulted in decreased estimates of resurgence half-life, however the estimated resurgence rates remained lower for the combined strategies compared to MDA alone (Additional file 1: Table S6). Increasing levels of case management decreased proportion of simulations where resurgence occurred (Additional file 1: Table S6).

### Vaccination to reduce years of mass deployment and reduce number of MDA rounds

Adding mass vaccination to MDA strategies of 2 years resulted in a similar or higher proportion of simulations with interruption of transmission in a shorter time period of intervention than compared to MDA deployed for 3 years. This was true when MDA and vaccination was administered simultaneously to the same people and when given independently from each other (random coverage between both interventions) (Table 1). For *Pf*PR_2–10_ = 2–4%, the proportion of simulations in which interruption of transmission occurred was 30% higher in strategies combining RTS,S-like-duration mass vaccination with full rounds of MDA for 2 years (65% transmission interruption) compared to 3 years of full MDA rounds only (50% transmission interruption) (Table 1). For MDA combined with longer duration vaccines, we estimated 80% of simulations interrupted transmission (Table 1). For MDA combined with a vaccine of lower initial efficacy (50%), we predicted 3 years of MDA to have greater chances to interrupt transmission.

**Table 1:** Percentage of simulations in which interruption of transmission occurred for different strategies and deploy times

Strategies	Interrupted transmission [%]			
	PfPR_2–10_ [%]			
	2	4	5	7
3 years full MDA rounds	80	20	0	0
2 years full MDA rounds + vaccine	90	40	0	0
	100^A^	40^A^	0^A^	0^A^
2 years full MDA rounds + longer duration vaccine	100	60	30	0
	80^A^	40^A^	30^A^	0^A^
2 years full MDA rounds + vaccine lower efficacy	60	0	0	0
	30^A^	0^A^	0^A^	0^A^
2 years full MDA rounds	20	0	0	0
2 years reduced MDA rounds + vaccine	70	10	0	0
	70^A^	0^A^	0^A^	0^A^
2 years reduced MDA rounds + longer duration vaccine	90	70	0	0
	80^A^	10^A^	0^A^	0^A^
2 years reduced MDA rounds + vaccine lower efficacy	30	0	0	0
	30^A^	0^A^	0^A^	0^A^

Percentage of resurgence in the simulations are shown for interventions (rows) at 60% coverage, for 2- and 3- years of full rounds of MDA, and 2 years of full or reduced rounds of MDA combined with mass vaccination with RTS,S like vaccine (initial efficacy against infection 91%, half-life 0.61 years), longer duration of protection vaccine (half-life 1.5 years), or lower initial efficacy (50%). Results are shown for different levels of initial *Pf*PR_2–10_ (%) (columns), and results where the vaccine is given to same proportion of the population than the simultaneous MDA are indicated with ^A^

Adding mass vaccination to MDA could replace 2 rounds of MDA in the second year for similar or improved chances of interruption of transmission, especially at lower prevalence levels. For *Pf*PR_2–10_ = 2% the proportion of simulations with interrupted transmission was 70% if 2 years of reduced rounds of MDA combined with mass vaccination was deployed (namely 3 rounds of MDA plus vaccination the first year and only 1 round of MDA plus vaccine the second year) compared to an interruption of transmission of 20% in strategies of 2 years of 3 rounds of MDA only (Table 1). Using a longer duration vaccine, 2 years of reduced rounds of MDA combined with mass vaccination at *Pf*PR_2–10_ = 2% led to a proportion of simulations with interrupted transmission up to 90% (Table 1). A vaccine with a lower initial efficacy of 50% could replace 2 rounds of MDA in the second year, but in both scenarios (vaccine and MDA with reduced rounds or full round of MDA) chances to interrupt transmission remained low (Table 1).

Simulations where MDA coverage was 60% but vaccine coverage was 40% in the combined interventions were additionally compared. In settings of *Pf*PR_2–10_ = 1–4% the proportion of simulations with transmission interruption was lower for vaccine coverage of 40% compared to 60%, thus increasing proportion with resurgence (Additional file 1: Table S3 and S4). However, even a strategy with a lower coverage of vaccination at 40%, combination strategies (mass vaccination and MDA) deployed during 2 years remained more favorable compared to using 3 years of MDA alone (Additional file 1: Table S3 and S4).

## Discussion

Given the enormous multiplicity of combinations of interventions that might be considered when targeting the whole population with combination strategies, and given the expense of mass deployment, it is critical to carry out in silico investigations of the potential of novel deployment options in order to avoid wasteful field trials of fruitless combinations [37].

To date little has been done to estimate the impact of adding a similar AIV to existing mass treatment strategies. By combining mass vaccination to treatment, the infectious reservoir is reduced by clearing infections in a large proportion of the population via MDA, and adding an intervention with ability to prevent infections for longer than the duration of the transmission season (even with waning protective immune responses restored with yearly immunizations) resulted in higher maximum prevalence reduction, increased chance of elimination, and importantly, delayed resurgence compared to interventions without the vaccine. Our simulations suggest that combining MDA with an AIV has a synergistic effect in regards to chances to interrupt transmission, and synergism increased with greater levels of initial prevalence and lower MDA coverage or case-management. Synergism can be the result of deploying two interventions which increases overall coverage of the population covered by at least one intervention, but as synergism was also predicted when the vaccine and the drug were given to the same people given coverage, although in a lesser extent, it is also likely a consequence of the interventions targeting different parasite life stages.

It will be important to consider whether the approaches modelled here are operationally feasible and what level of health system and program strength is required to coordinate vaccination and drug campaigns. For this reason, we investigated a range of coverages and our main results were presented for coverages of 60%, assuming any higher was too optimistic. In those conditions, with initial *Pf*PR_2–10_ = 4%, we found that for the simulations where transmission is not interrupted, the rate of resurgence is halved compared to MDA alone, extending the resurgence half-life up to two and a half years. Alternatively, we investigated a higher MDA coverage compared to vaccination and we found that adding mass vaccination even at a lower coverage (40%) remained beneficial in delaying resurgence and reducing MDA rounds. We also found limited differences in impact between the combined strategies with vaccination before or during the transmission season, arguing for a preference of during the season with 3 visits as opposed to more visits.

In addition, we estimated that vaccines of longer duration of protection can further delay resurgence, arguing that research into the feasibility of increasing longevity of protection of a vaccine has importance for the elimination strategies investigated here.

The addition of mass vaccination to MDA, compared with MDA alone, has the potential to reduce the number of MDA rounds needed, as well as the number of years that interventions may be needed, to achieve similar public health benefits of prevalence reduction and chances to interrupt transmission, and thus reduce the length of deployment of interventions from 3 to 2 years. Reducing the length of overall intervention deployment has the potential to reduce the number of mass intervention visits, and thus costs of visits. Moreover, although an economic analysis was outside the scope of this study, a shorter period of deployment could be beneficial to national malaria control and elimination strategies facing budgetary constraints. Although this work did not explicitly explore mobility, we did model importations of infections and highly mobile populations will limit operational coverage of the vaccines and MDA. Limited duration mass interventions are not intended to tackle importation. As with MDA, importation is likely to have little effect on the estimated prevalence reduction unless initial prevalence is very low, and in these settings where interruption of transmission is achieved a high level of case management will be important to maintain zero prevalence [16]. In line with this, we found importation of infections had little effect on prevalence reduction achieved but that time to resurgence decreased as importation increased.

Furthermore, interruption of transmission with mass interventions is likely only possible for scenarios of low initial prevalence (low prevalence as a result of vector control or other interventions), with a high level of case management and an increase in other intervention tools, which was recently highlighted by the WHO Malaria Policy Advisory Committee meeting with regards to the use of MDA [38]. Our simulations also indicated that, chances to interrupt transmission increase with higher case management. This implies that such mass interventions should not be investigated independently of case management, and that together with vector control or reactive tools, will play a decisive role in the success of the interventions to reach elimination and avoid resurgence.

This work was confined to a limited number of model-based scenarios of mass interventions and as such there are several limitations. Firstly, several aspects not captured might enhance or diminish the impact of mass vaccination combined with MDA. On a biological level, vaccination with an AIV with drugs has not been sufficiently investigated, and it is unclear how vaccine efficacy, immune dynamics in response to the vaccine, the length of protection, or the rate of vaccine failure would be influenced. Such effects are unknown, however a lower rate of vaccine failure and longer duration of protection would delay resurgence and extend the pre-elimination period, and conversely higher failure rates would decrease benefits. Additionally, we did not address any potential evolutionary effects such as vaccine insensitivity, nor antigenic sin. However, we note deploying with treatment may protect against evolution of vaccine insensitivity.

Secondly, the underlying vaccine assumptions in the models, including the effect of a forth vaccine dose, may influence results. Vaccine properties were based on analysis of the results of RTS,S Phase 3 clinical trials [13], which targeted infants aged 6 to 12 weeks and young children aged 5 to 17 months. In the context of mass vaccination, as the efficacy and protection might be different for different ages and previous exposure, extension of the protection profile and vaccine parameterization from children to the entire population is a necessary simplification based on currently available data. A full sensitivity analysis around vaccine efficacy profiles has not been performed in this study, but simulations where initial efficacy against infection for children and adults was reduced to 50% suggest that this would considerably decrease the prevalence and case management settings where the combination strategies would reach very low transmission levels or interruption of transmission.

Thirdly, we did not model potential increases in vector control or case management levels over the time of intervention, either due to vector control campaigns or active efforts to improve access to treatment or due to a better efficiency in the health system as fewer cases need to be treated. Both would further reduce prevalence levels following mass intervention deployment, and in some settings, would likely enhance the effect of mass vaccination, especially during the resurgence period. Conversely, we did not model deceasing levels of vector control which may decrease the benefits of the mass intervention strategies or increase rates of resurgence.

We chose a 3-month seasonal malaria transmission setting of sub-Sahel African countries approximating the pattern in Senegal where SMC is ongoing, and investigated the impact on average transmission, not accounting for heterogeneities in transmission. Further studies could be undertaken to tailor the analysis specifically to a country or region, with corresponding transmission pattern, heterogeneity, and other underlying settings, such as case management or adapted timing and duration of deployment. The impact of interventions may be sensitive to timing and the seasonal pattern chosen for malaria transmission, and that the heterogeneity of transmission across a given region will imply a need for focal planning.

Previous modeling has shown the impact of MDA is very sensitive to population size, being best suited for small populations and with total impact sensitive to the proportion of the population receiving no treatment from any of the deployed rounds [39]. In our current work, individual rounds of MDA were given to a random proportion of the population for a given coverage, mass vaccination was deployed either independently to MDA or in some settings simultaneously to MDA, and the 3 vaccine doses independently from the fourth dose. As expected, scenarios with total correlation between those covered by MDA and mass vaccination predicted a slightly reduced impact, but the key determinants probably remains in the random coverage between MDA rounds similar to results found for multiple rounds of MDA [16], and total correlation between rounds has not been investigated here. In more realistic settings the proportion of the population covered would neither be completely independent nor would it be exactly the same between deployments, and thus our assumptions might be slightly optimistic so that the nominal coverages we simulate reflect higher values in the field.

Given that MDA and SMC are currently implemented in several settings [19, 40], and that the combination of RTS,S and SMC is being tested in field trials [14], we are in a position to consider adapting these combination of interventions to different ages. Technical challenges however remain. It is envisioned that RTS,S may be delivered in much the same way as meningococcal A conjugate vaccine is being deployed in sub-Saharan Africa in 1–29 year old, initially in intense mass vaccination campaigns, and then followed by introduction into existing Extended Immunization Programmes in the individual countries [41]. Any vaccines used in malaria elimination campaigns will need to be WHO pre-qualified ensuring that characteristics are aligned with cold-chain requirements available in the countries where it is deployed [42]. For an AIV to be used as a mass intervention in Africa or Asia, several important unknowns must be addressed with respect to immunogenicity in adults and immunogenicity in populations outside Africa. Measurement of immune responses and protection in adults have been tested in the Mekong, where RTS,S was administered alone or in combination to drugs [43] as are many other CHMI (controlled human malaria infection) studies of RTS,S, R21, and PfSPZ. These studies should provide additional evidence that could be used to model their potential role in mass vaccination scenarios. Other challenges include safety and efficacy testing when vaccines are administered with drugs and regulatory hurdles for use of any of the AIVs for children and adults, which may be more difficult with new antigens. From an operational perspective, the challenges may be significant, ensuring sufficient coverage of the first three or required initial number of doses of the vaccine. And lastly, to make use of the potential benefit in delaying resurgence, settings must be prepared with additional interventions during the low prevalence period following 2 or 3 years of mass intervention, and as with MDA, it will be important to include surveillance and response strategies combined with strong health systems to address importation of infection and preserve this delay in resurgence.

Given combining mass vaccination with treatment has potential to delay resurgence for longer than treatment alone, there may be a role in malaria outbreaks or in fragile contexts where health systems are weakened by other disease outbreaks [44] or conflict. However, the number of doses required to reach high efficacy may limit this application. There may also be a role in helping mitigate spread of drug resistance in the Mekong or for targeting forest workers [37], or for areas in which residual or outdoor transmission renders indoor residual spraying less effective, but further investigations are required.

Our modelling study provides evidence of potential use of an AIV in malaria elimination with characteristics similar to the most advanced vaccines: high initial efficacy and with limited duration of protection in an area with highly seasonal malaria transmission. Assumptions in regards to demographics, past history of malaria, transmission profile, vector species, and health systems strengths were not geographic specific. An important next step will be to understand which settings and countries these mass interventions strategies are feasible for and what range of settings with pockets of focal transmission. This would include an assessment of cost-effectiveness.

## Conclusion

This work reports modelling outputs of vaccination with existing and potential forthcoming AIVs combined with MDA - indicating such a combination could be a possible strategy to rapidly decrease prevalence and keep prevalence levels lower than would MDA alone for several years before resurgence, thus challenging thinking on the role of malaria tools already at our disposal. A deep understanding of current malaria burden and level of case management, with a cautious investigation of the timing and operational feasibility of the mass interventions would nevertheless be of paramount importance before implementing such a strategy for malaria elimination, as the risk of resurgence and thus failure of the mass campaigns will remain a threat. Acknowledging that malaria elimination will not be achieved with a single intervention, we should continue to investigate combination intervention strategies targeting different parts of the lifecycle that will help achieve our goals.

## Supplementary information

**Additional file 1: Table S1.** Overview of the input parameter values for the simulations and the direct outputs measured. **Table S2.** Overview of the main and supplementary simulated strategies. **Figure S1**. Single simulation examples of estimated continuous all age prevalence following different intervention. **Figure S2.** Median and range of estimated yearly average all age prevalence following different intervention strategies. **Figure S3.** Relative impact of combined strategies for different coverage levels of each intervention. **Figure S4.** Relative maximum prevalence reached depending on intervention coverage. **Figure S5.** Impact of mass vaccination compared to MDA with same coverage levels comparing strategies 3 and 4 with strategy 1 and 2. **Table S3.** Risk of resurgence [%] for different deployment strategies. **Table S4.** Risk of resurgence [%] for different deployment strategies, with reduced coverage of mass vaccination. **Table S5.** Predicted risk of resurgence [%] for different strategies at low prevalence levels (*Pf*PR_2–10_ 1 to 5%) for different levels of case management and intervention coverage. **Figure S6.** Interruption of transmission for different strategies with lower case management. **Figure S7.** Interruption of transmission and synergism for different combined strategies with simultaneous MDA and mass vaccination interventions delivered to the same proportion of the population given coverage. **Figure S8.** Interruption of transmission for mass vaccination and combined strategies with lower vaccine efficacy. **Figure S9**. Synergy coefficient between mass vaccination and mass drug administration in the probability to interrupt transmission. **Table S6.** Estimated resurgence parameters for 2 years deployment of MDA, mass vaccination, or combination of both MDA and mass vaccination, including 2 rates of imported infections. **Figure S10.** Relationship between entomological inoculation rate, EIR, and effective access to care, E_14_, with PfPR_2–10_ and prevalence in all population.

## Data Availability

The simulation datasets generated and analyzed during the current study are available from the corresponding author on request.

## Abbreviations

AIV: Anti Infective Vaccine
E_14_: 14 day effective treatment coverage
EIR: Entomological Inoculation Rate
MDA: Mass Drug Administration
*Pf*PR_2–10_: Initial parasite prevalence levels in 2–10 year olds
SMC: Seasonal Malaria Chemoprevention
