# Supplementary material for: Mass campaigns combining antimalarial drugs and anti-infective vaccines as seasonal interventions for malaria control, elimination and prevention of resurgence: a modelling study
Source: BMC Infect Dis. 2019 Oct 29;19:920. doi: 10.1186/s12879-019-4467-4 (PMC6820916; doi:10.1186/s12879-019-4467-4)
Supplement: Supplementary file 1 — Additional file 1: Table S1. Overview of the input parameter values for the simulations and the direct outputs measured. Table S2. Overview of the main and supplementary simulated strategies. Figure S1. Single simulation examples of estimated continuous all age prevalence following different intervention. Figure S2. Median and range of estimated yearly average all age prevalence following different intervention strategies. Figure S3. Relative impact of combined strategies for different coverage levels of each intervention. Figure S4. Relative maximum prevalence reached depending on intervention coverage. Figure S5. Impact of mass vaccination compared to MDA with same coverage levels comparing strategies 3 and 4 with strategy 1 and 2. Table S3. Risk of resurgence [%] for different deployment strategies. Table S4. Risk of resurgence [%] for different deployment strategies, with reduced coverage of mass vaccination. Table S5. Predicted risk of resurgence [%] for different strategies at low prevalence levels (PfPR2–10 1 to 5%) for different levels of case management and intervention coverage. Figure S6. Interruption of transmission for different strategies with lower case management. Figure S7. Interruption of transmission and synergism for different combined strategies with simultaneous MDA and mass vaccination interventions delivered to the same proportion of the population given coverage. Figure S8. Interruption of transmission for mass vaccination and combined strategies with lower vaccine efficacy. Figure S9. Synergy coefficient between mass vaccination and mass drug administration in the probability to interrupt transmission. Table S6. Estimated resurgence parameters for 2 years deployment of MDA, mass vaccination, or combination of both MDA and mass vaccination, including 2 rates of imported infections. Figure S10. Relationship between entomological inoculation rate, EIR, and effective access to care, E14, with PfPR2–10 and prevalence in all population. [file 12879_2019_4467_MOESM1_ESM.pdf]

Additional File 1 for:

Mass campaigns combining antimalarial drugs and anti-infective vaccines as seasonal interventions for malaria control, elimination and prevention of resurgence: a modelling study

Authors:

Flavia Camponovo[1,2], Chris F. Ockenhouse[3], Cynthia Lee[3], Melissa A. Penny*[1,2]

[1] Swiss Tropical and Public Health Institute, Basel, Switzerland

[2] University of Basel, Basel, Switzerland

[3] PATH's Malaria Vaccine Initiative, Washington, DC, USA

Corresponding author: melissa.penny@unibas.ch

This PDF file includes:

Figs. S1 to S8
Tables S1 to S7
References for SI reference citations

Table S1: Overview of the input parameter values for the simulations and the direct outputs measured

Parameter	Values	Comments
**Model Specificity**	Model R0068 (1)	Heterogeneity in transmission: within host variability. No immunity decay
**EIR and *Pf*PR$_{2-10}$**	EIR = 0.5, 1, 2, 3, or 5<br>Corresponding to *Pf*PR$_{2-10}$ 1-20%	*Pf*PR$_{2-10}$ range of approximately 1-20%, see supplementary Figure S1
**Imported infections [per 1000 population]**	0, 2, 20	Based on values used in previous simulations, reflecting a range of realistic importation of infections (2)
**Level of case management as 5-day effective treatment probabilities [%]**	5, 10, 20, 30, 40, 50%	This equates to 14 day effective coverage (E_{14}) of 15, 25, 45, 60, 70, 80%, chosen to reflect a range of current and realistic levels as well as improved levels of case management
**MDA drug parameterization**	Dihydroartemisinin piperaquine (DHAp)<br>Initial blood stage clearance efficacy of 100%, with half-life of chemoprevention of 12 days	Parameterization as previously used in MDA modelling studies (3)
**Vaccine parameterization (refered to as RTS,S-like-duration vaccine)**	initial efficacy = 91%<br>Biphasic decay implemented via a Weibull decay function (k=0.69) and half-life = 0.61 years (7.3 months)	As per previously modelling of RTS,S with underlying vaccine properties parameters (4)<br>The fourth and fifth dose is assumed to have same parametrization
**Longer duration vaccine parameterization**	initial efficacy = 91%<br>implemented via a Weibull decay function (k=0.69) and half-life = 1.5 years	
**Lower efficacy vaccine**	initial efficacy = 50%<br>implemented via a Weibull decay function (k=0.69) and half-life = 0.61 or 1.5 years	
**Coverage of interventions [%]**	0, 30, 40, 60, 70, 80, 90, 100%	Includes realistic levels, but also out-of-range levels for a better understanding of the vaccine – MDA interactions and relative benefits
**Congruency between interventions and covered population**	The 3 vaccine doses are given to the same population (given coverage)[1], but the fourth and the fifth dose are given to random population<br>MDA is given to random proportion of the population (given coverage) for each round; and independent from vaccination[2] unless otherwise specified[3]	[1] assuming 100% adherence to the 3 immunization doses<br>[2] both when the vaccine is delivered before or simultaneously to the MDA rounds<br>[3] in a subset of the simulations, vaccination and MDA are delivered simultaneously to the same proportion of the population, given coverage
**Target age of mass vaccination**	Minimum age is 9 months for third dose, with first dose from 5 months of age	Minimum age of 5 months at first vaccination is assumed, as intended following RTS,S implementation (5)
**Target age from MDA**	All ages but with minimum age from 6 months of age	As defined previously (3), pregnant women were not explicitly excluded but would reflect lower coverages
**Population size**	10’000	MDA has shown to have better efficacy in targeted elimination strategies of small populations (6)
**Monitored outputs from the simulations**	Number of patent infections per year	
**Definition of transmission interruption for each simulation**	On average across the 5-10 years post intervention deployment period, less than 1 infected individual in 10’000	

Table S2: Overview of the main and supplementary simulated strategies. The strategies include: MDA alone (strategies 1 and 2), vaccine alone (strategies 3, 4, s3, s4), or MDA with vaccine (strategies 5 to 8, and s5 to s8). MDA application alone is 3 rounds coinciding with the pattern of seasonal transmission, with 2-3 years of 3 rounds or 3 rounds for only the first year followed by 1-2 years of 1 round at the beginning of the transmission season (strategy 1 and 2); RTS,S-like-duration vaccine or longer duration vaccine application alone is 3 rounds coinciding with the pattern of seasonal transmission with 1-2 years of 1 dose at the beginning of the season (strategy 3 and 4) or as 3 rounds before the pattern of seasonal transmission, with 1-2 years of 1 dose at the beginning of the season (strategy s3 and s4); and strategies combining MDA with RTS,S-like-duration vaccine or longer duration vaccine are a combination of all MDA and vaccine implementations combined together (strategies 5 to 8 and s5 to s8).

strategy	MDA 3 rounds during the transmission season	MDA 1 round at start of transmission season	RTS,S-like-duration vaccine; 3 rounds during transmission season	RTS,S-like-duration vaccine; 1 round start of transmission season	Longer duration vaccine; 3 rounds during transmission season	Longer duration vaccine; 1 round start of transmission season
control	-	-	-	-	-	-
1	2 to 3 years	-	-	-	-	-
2	year 1	year 2 or year 2 and 3	-	-	-	-
3	-	-	year 1	year 2 or year 2 and 3	-	-
4	-	-	-	-	year 1	year 2 or year 2 and 3
5	2 to 3 years	-	year 1	year 2 or year 2 and 3	-	-
6	year 1	year 2 or year 2 and 3	year 1	year 2 or year 2 and 3	-	-
7	2 to 3 years	-	-	-	year 1	year 2 or year 2 and 3
8	year 1	year 2 or year 2 and 3	-	-	year 1	year 2 or year 2 and 3
**supplement strategy**	**MDA 3 rounds during the transmission season**	**MDA 3 rounds during the transmission season**	**RTS,S-like-duration vaccine; 3rd dose at the start of transmission season**	**RTS,S-like-duration vaccine; 1 round start of transmission season**	**Longer duration vaccine; 3rd dose at the start of transmission season**	**Longer duration vaccine; 1 round start of transmission season**
s3	-	-	year 1	year 2 or year 2 and 3	-	-
s4	-	-	-	-	year 1	year 2 or year 2 and 3
s5	2 to 3 years	-	year 1	year 2 or year 2 and 3	-	-
s6	year 1	year 2 or year 2 and 3	year 1	year 2 or year 2 and 3	-	-
s7	2 to 3 years	-	-	-	year 1	year 2 or year 2 and 3
s8	year 1	year 2 or year 2 and 3	-	-	year 1	year 2 or year 2 and 3

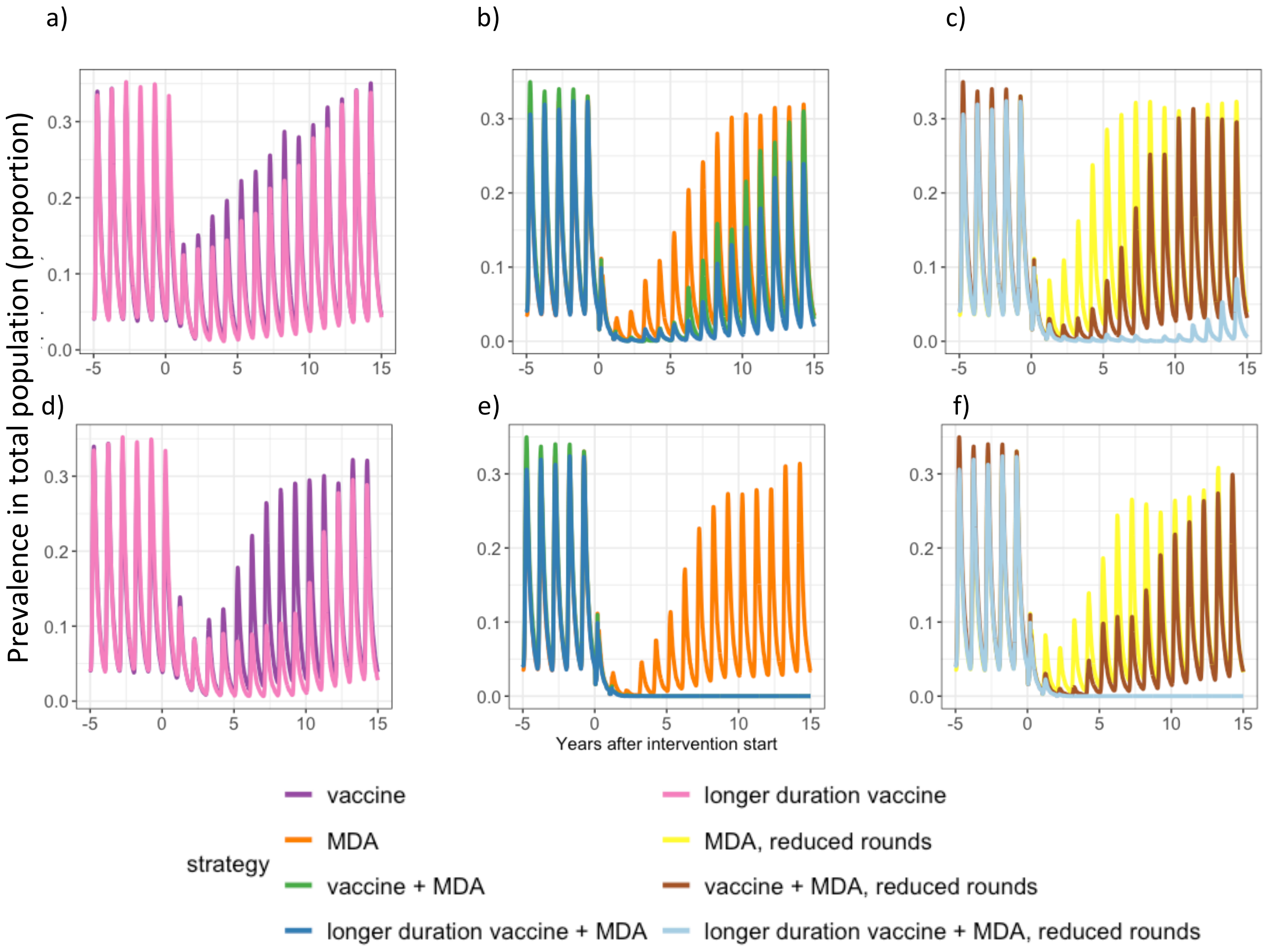

Figure S1: Single simulation examples of estimated continuous all age prevalence following different intervention. Plots (**a-c**) are for 2 years of mass intervention and plots (**d-e**) for 3 years of mass intervention. (**a**) and (**d**): estimated all age prevalence following mass vaccination with RTS,S-like-duration vaccine (purple) or mass vaccination with longer duration vaccine (pink), (**b**) and (**e**) estimated all age prevalence following full rounds of MDA alone (orange) or in combination with mass vaccination with RTS,S-like-duration vaccine (green) or mass vaccination with longer duration vaccine (blue), (**c**) and (**f**) estimated all age prevalence following reduced rounds of MDA alone (yellow) or in combination with mass vaccination with RTS,S-like-duration vaccine (brown) or mass vaccination with longer duration vaccine (light blue). Intervention coverage was assumed at 60%, with an initial yearly average *Pf*PR$_{2\text{-}10}$ ≈ 3% – 4% with peak *Pf*PR$_{2\text{-}10}$ ≈ 10% –15% (corresponding to an initial EIR of 2 and effective access to care E_{14}=45%). Simulations were chosen at random, full variation of predictions for each strategy are shown in Figure S3.

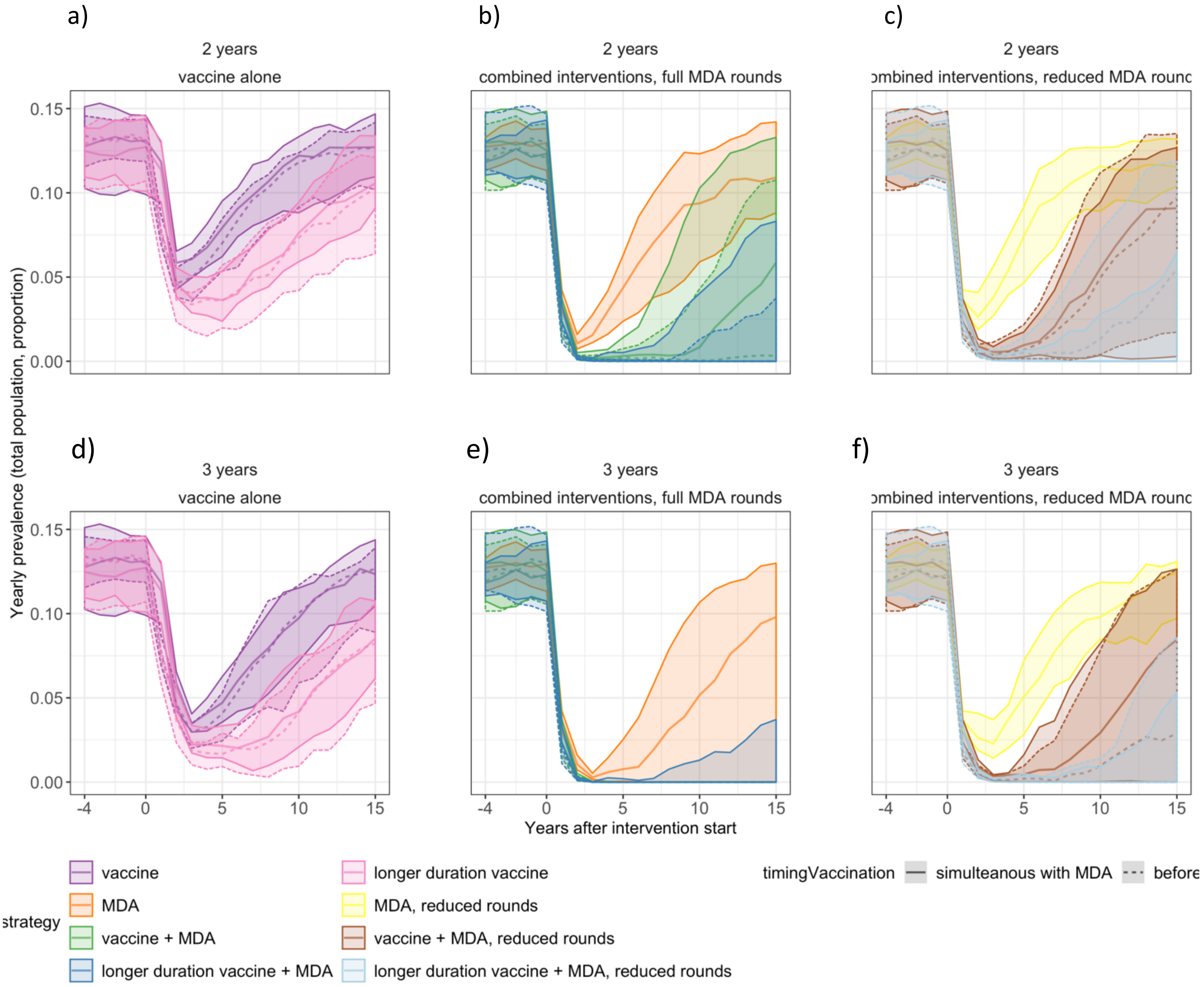

Figure S2: Median and range of estimated yearly average all age prevalence following different intervention strategies. Plots (**a-c**) are for 2 years of mass intervention and plots (**d-e**) for 3 years of mass intervention. (**a**) and (**d**): estimated all age prevalence following mass vaccination with RTS,S-like-duration vaccine (purple) or mass vaccination with longer duration vaccine (pink), (**b**) and (**e**) estimated all age prevalence following full rounds of MDA alone (orange) or in combination with mass vaccination with RTS,S-like-duration vaccine (green) or mass vaccination with longer duration vaccine (blue), (**c**) and (**f**) estimated all age prevalence following reduced rounds of MDA alone (yellow) or in combination with mass vaccination with RTS,S-like-duration vaccine (brown) or mass vaccination with longer duration vaccine (light blue). Strategies where vaccination was performed before the transmission season are represented by dashed lines. Each intervention is represented by the median and minimum-maximum range across 10 simulations per a strategy. Intervention coverage was assumed at 60%, with initial average annual *Pf*$PR_{2\text{-}10}$ ≈ 3% – 4% with peak *Pf*$PR_{2\text{-}10}$ ≈ 10% –15% (corresponding to an initial EIR=2 and effective access to care E_{14}=45%).

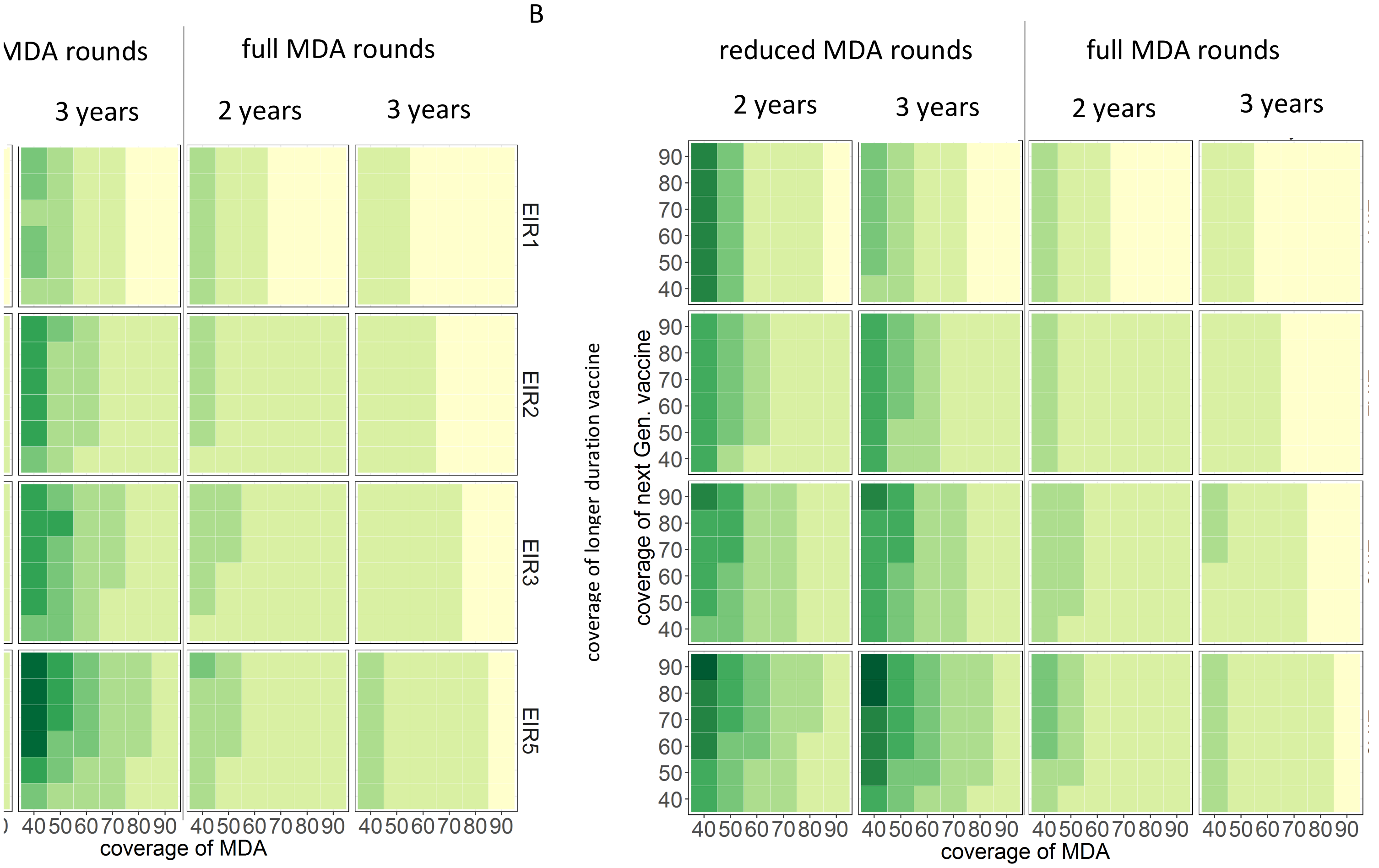

e impact of combined strategies for different coverage levels of each intervention. The x-axis indicates the coverage c ıss vaccination. Colour represents the impact calculated as the relative maximum prevalence reduction of the combined inter prevalence reduction when using MDA alone (strategy 2) at the same coverage levels (relative impact =1 means that th ıe same impact, and a level of 2 means that mass vaccination with MDA is 2 times greater than MDA alone). **A** Represen ˈS,S-like-duration vaccine and **B** represents the impact of mass vaccination with longer duration vaccine. From left to write, res educed MDA rounds with mass vaccination for 2 years, the relative impact of reduced MDA rounds with mass vaccination fo rounds with mass vaccination for 2 years and the relative impact of full MDA rounds with mass vaccination for 3 years. From to 3 and 5.

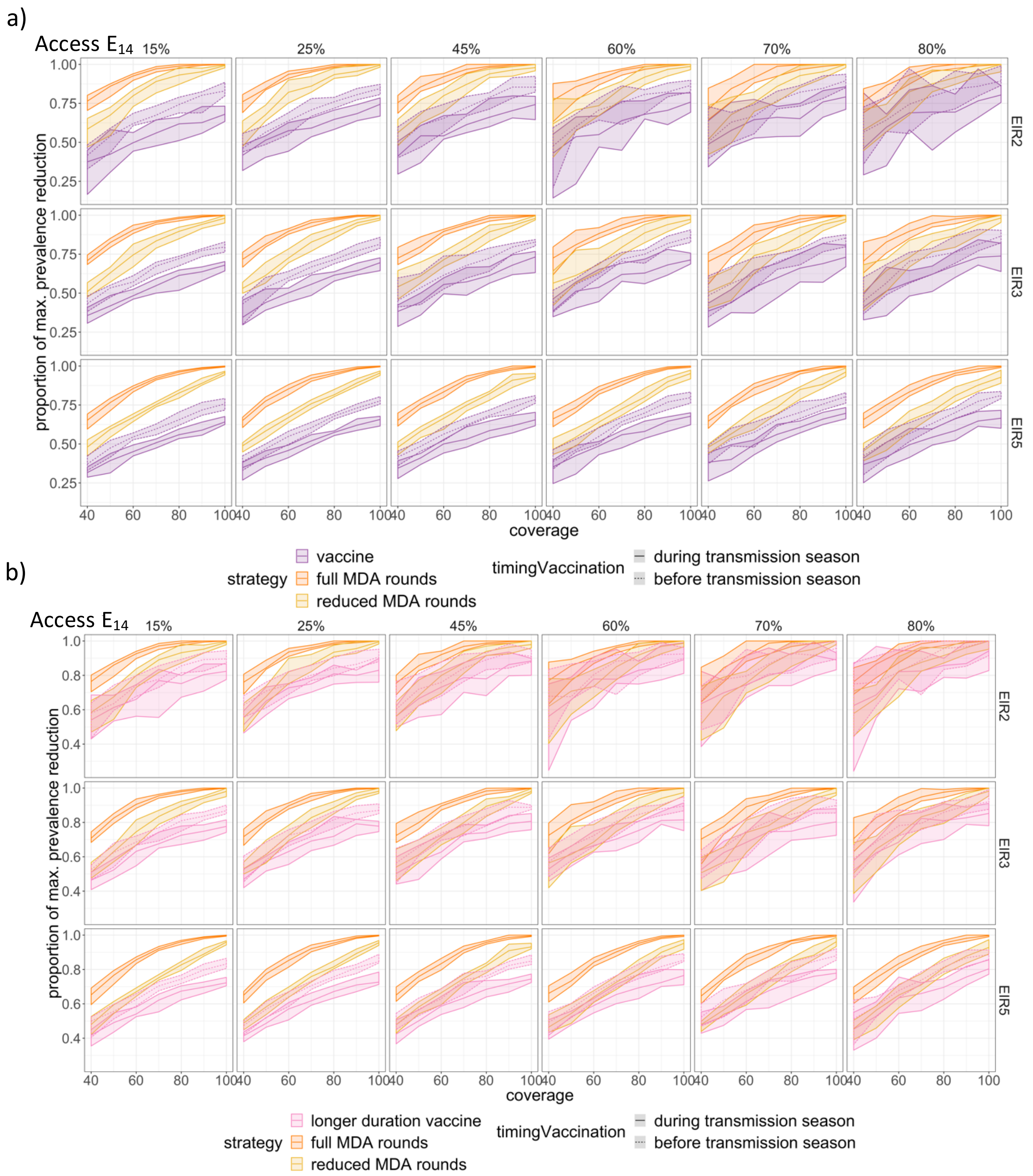

Figure S4: Relative maximum prevalence reached depending on intervention coverage. Maximum prevalence reduction reached by mass vaccination before (dashed lines) or during (solid lines) the transmission season and full (in orange) or reduced (in yellow) rounds of MDA. **a)** MDA is compared to mass vaccination with RTS,S-like-duration vaccine (purple) and **b)** MDA is compared to mass vaccination with longer duration vaccine (pink). The maximum prevalence reduction of each strategy in shown for different coverage levels on the x-axis, maximum prevalence reduction is shown on the y-axis from 0 (no prevalence reduction) to 1 (prevalence = 0). Each intervention is represented by the median and maximum-minimum across 10 simulations. Results are shown for different levels of passive case management (14-day effective treatment probabilities, columns) and different levels of EIR (rows), and the strategies are deployed for 2 years.

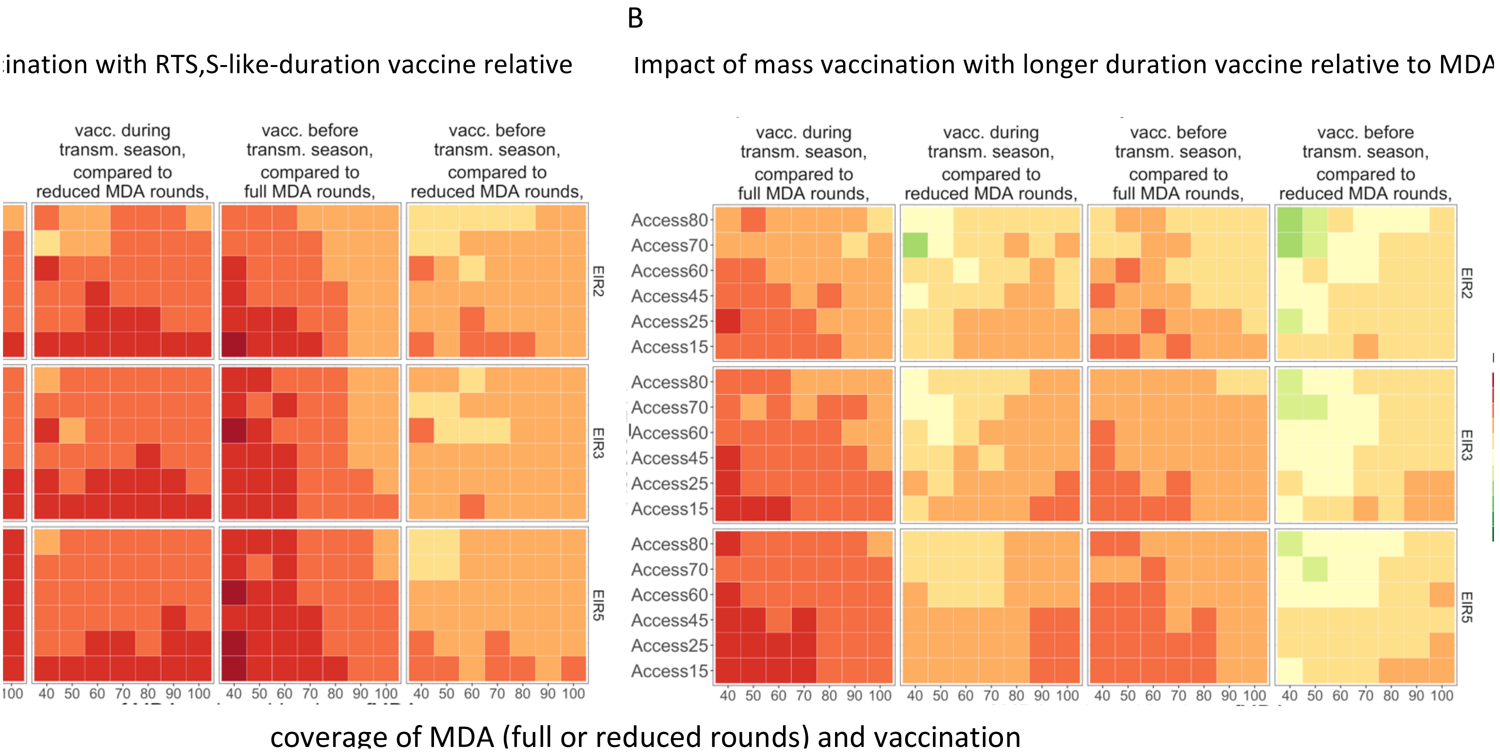

ass vaccination compared to MDA with same coverage levels comparing strategies 3 and 4 with strategy 1 and 2. The x-a mass vaccination and MDA, and the y-axis indicates the passive case management levels (14-day effective treatment proba relative impact estimated as the relative maximum case reduction achieve by mass vaccination normalized by the estima ore green (more positive) the higher the impact predicted by the simulations of mass vaccination compared to MDA, and th igher the impact of MDA compared to mass vaccination. **A** Represents the impact of mass vaccination with RTS,S-like-dura impact of next generation mass vaccination with an increased duration of protection (half-life 1.5 years). Results are shown full rounds of MDA and with reduced rounds of MDA, and for mass vaccination deployed before and during the transmission

resurgence [%] for different deployment strategies. The probability of resurgence is given by the percentage of
e interruption of transmission did not occur. The simulations include simulations in both scenarios where the interve
s. Coverage of both vaccine and MDA deployment are 60%, and E_{14} = 45%. Strategy numbers are defined as in Ta

me eployment	EIR	E_{14}	$PfPR_{2-10}$	Strategies												
				1	2	3	4	5	6	7	8	s3	s4	s5	s6	s7
**ears**	0.5	45	<1	20	40	70	30	0	0	0	10	60	30	0	0	0
**ears**	1	45	2	80	90	100	80	10	30	0	10	100	70	10	40	10
**ears**	2	45	4	100	100	100	100	60	90	40	30	100	100	50	90	10
**ears**	3	45	5	100	100	100	100	100	100	70	100	100	100	70	100	20
**ears**	5	45	7	100	100	100	100	100	100	100	100	100	100	100	100	100
**ears**	0.5	45	<1	0	40	80	10	0	10	0	0	50	20	0	0	0
**ears**	1	45	2	20	70	70	80	0	10	0	0	100	70	0	20	0
**ears**	2	45	4	80	100	100	100	0	90	10	40	100	100	0	50	0
**ears**	3	45	5	100	100	100	100	20	90	10	70	100	100	30	70	0
**ears**	5	45	7	100	100	100	100	80	100	30	100	100	100	60	100	20

resurgence [%] for different deployment strategies, with reduced coverage of mass vaccination. The probab
ntage of simulations in each setting (n=10) where interruption of transmission did not occur. The simulations include
e interventions are deployed during 2 and 3 years. Coverage of MDA deployment are 60% and mass vaccination 4
are defined as in Table S2.

Time deployment	EIR	E_{14}	$PfPR_{2-10}$	Strategies											
				3	4	5	6	7	8	s3	s4	s5	s6	s7	s8
**2years**	0.5	45	<1	60	3	0	0	0	0	50	50	0	0	0	2
**2years**	1	45	2	100	90	30	70	10	30	100	90	10	50	40	40
**2years**	2	45	4	100	100	60	100	70	90	100	100	40	100	60	100
**2years**	3	45	5	100	100	90	100	100	90	100	100	100	100	80	100
**2years**	5	45	7	100	100	100	100	100	100	100	100	100	100	100	100
**3years**	0.5	45	<1	50	30	10	10	0	0	30	50	0	0	0	0
**3years**	1	45	2	100	80	10	20	0	40	100	90	0	10	10	0
**3years**	2	45	4	100	100	10	90	30	60	100	100	10	80	20	50
**3years**	3	45	5	100	100	50	100	30	80	100	100	40	100	10	100
**3years**	5	45	7	100	100	100	100	50	100	100	100	90	100	70	100

Table S5: Predicted risk of resurgence [%] for different strategies at low prevalence levels (*Pf*PR$_{2\text{-}10}$ 1% to 5%) for different levels of case management and intervention coverage. The probability of resurgence is estimated as the percentage of simulations in each strategy (n=20) where interruption of transmission did not occur. The simulations include simulations where initial prevalence levels range between 1%-5% given the three different case management levels E_{14} = 25%; 45% and 60%, and in both scenarios where the interventions are deployed during 2 and 3 years. Coverage of both vaccine and MDA deployments was 60%. Strategy numbers are defined as in Table S2

**Time of deployment**	**Strategies**													
	**1**	**2**	**3**	**4**	**5**	**6**	**7**	**8**	**s3**	**s4**	**s5**	**s6**	**s7**	**s8**
	E_{14} = 25%													
2 years	100	100	100	100	85	70	25	30	100	100	55	65	10	35
3years	40	100	100	95	20	65	0	15	100	90	10	50	0	20
	E_{14} = 45%													
2 years	90	95	100	90	35	60	20	20	100	85	30	65	10	45
3years	50	85	85	90	0	50	5	20	100	85	0	35	0	10
	E_{14} = 60%													
2 years	80	90	95	85	35	55	5	30	95	100	35	65	5	15
3 years	50	85	95	85	5	20	0	15	85	70	0	40	0	0

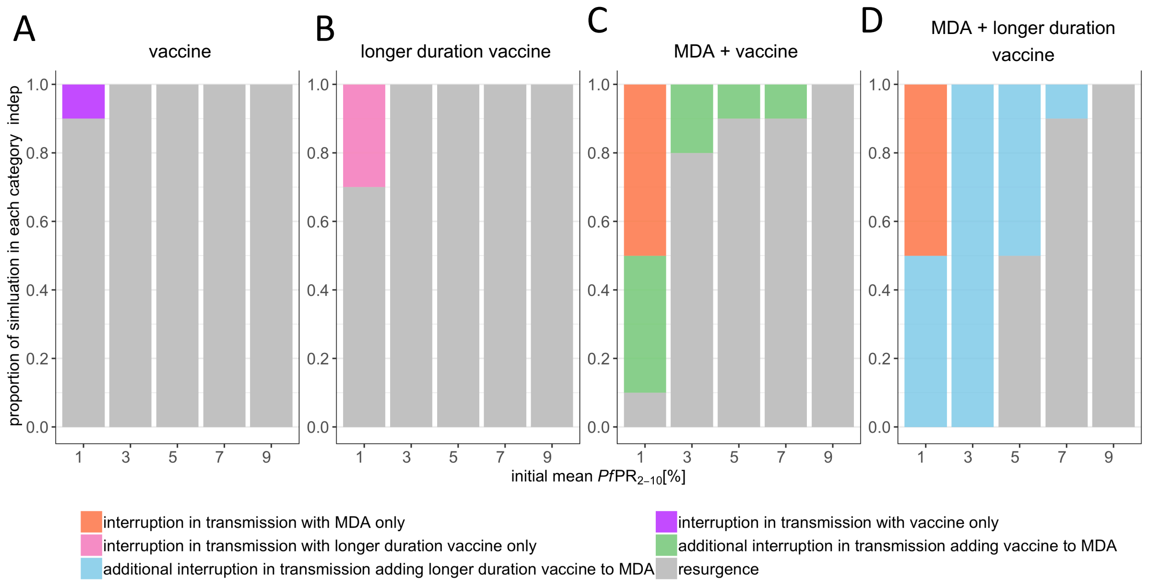

Figure S6: Interruption of transmission for different strategies with lower case management. Proportion of simulations in which interruption of transmission is estimated to be achieved with **A** mass vaccination, **B** mass vaccination with longer duration of protection, and MDA combined with **C** mass vaccination or **B** longer duration. Initial *Pf*PR$_{2\text{-}10}$ (%) levels are shown on the x-axis, and proportion of the simulations falling into each category are shown on the y-axis. All interventions were deployed for two years at a coverage of 60%, and the underlying effective access to care, E_{14}, was 25%. Categories of simulations are *i)* interruption of transmission occurred with single interventions, namely with mass vaccination with RTS,S like vaccine (purple) or longer duration vaccine (pink), or with MDA (orange), *ii)* interruption of transmission occurred only adding mass vaccination to MDA (green and blue using with RTS,S like vaccine or longer duration vaccine respectively), and *ii)* resurgence occurred and no interruption of transmission was achieved (grey).

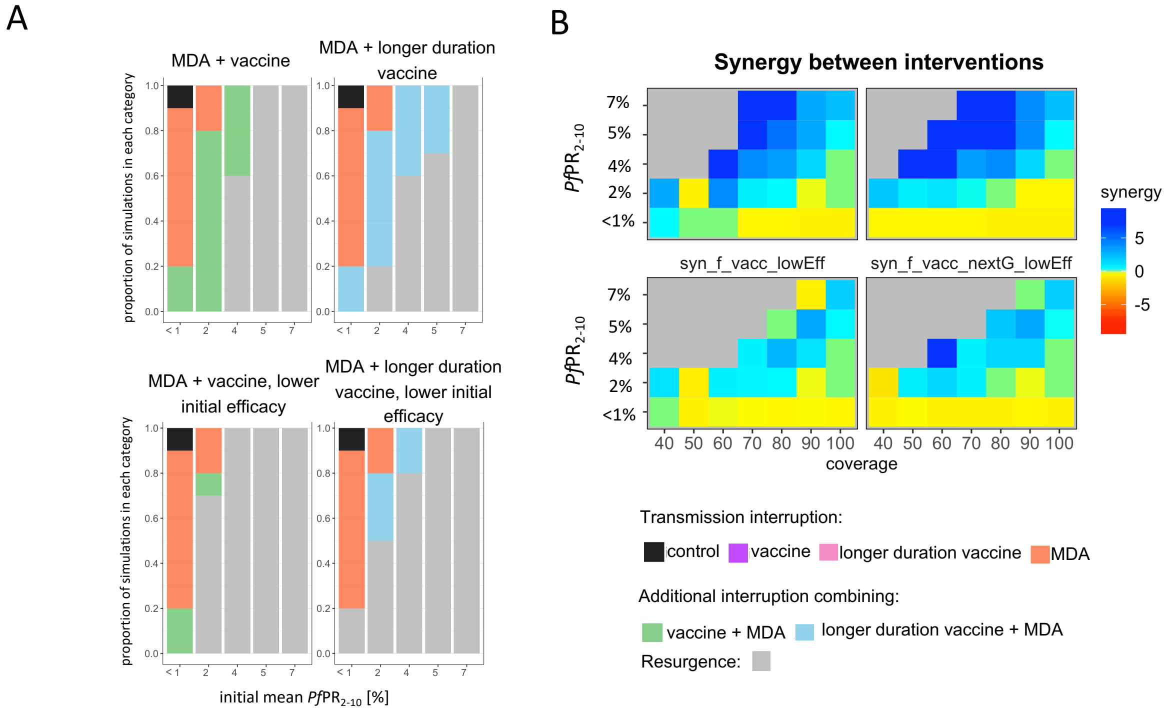

Figure S7: Interruption of transmission and synergism for different combined strategies with simultaneous MDA and mass vaccination interventions delivered to the same proportion of the population given coverage. A Proportion of simulations in which interruption of transmission is estimated to be achieved with mass vaccination combined to MDA. Upper plots indicate interruption of transmission using a vaccine with initial efficacy of 91% and half-life of 0.61 years (upper row, left) or 1.5 years (upper row, right), and lower plots indicate interruption of transmission using a vaccine with initial efficacy of 50% and half-life of 0.61 years (bottom row, left) or 1.5 years (bottom row, right). Initial *Pf*PR$_{2\text{-}10}$ (%) levels are shown on the x-axis, and proportion of the simulations falling into each category are shown on the y-axis. All interventions were deployed for two years at a coverage of 60%. Categories of simulations are *i)* interruption of transmission occurred with no intervention at all, due to very low initial prevalence (black), *ii)* interruption of transmission occurred with single interventions, namely with mass vaccination with RTS,S like vaccine or lower efficacy (purple) or longer duration vaccine (pink), or with MDA (orange), *iii)* interruption of transmission occurred only adding mass vaccination to MDA (green and blue using with RTS,S like vaccine or longer duration vaccine respectively), and *iv)* resurgence occurred and no interruption of transmission was achieved (grey).**B** Estimated synergy coefficient (σ) of the combined mass vaccination and MDA intervention in regards probability to interrupt transmission. The x-axis indicates coverage levels of bot MDA and mass vaccination, and the y-axis initial *Pf*PR$_{2\text{-}10}$ (%), and the level of synergy between the two intervention strategies are indicated by colour. Blue represents synergistic behavior (>0) in the combined MDA and mass vaccination, light green represents values of 0 which imply the combined interventions are not more than additive, and colours yellow to red represent values less than 0 which implies less than additive or maximum level was reached by one or both single interventions. Grey areas represent settings where resurgence occurred in all simulation, thus no synergy could be calculated. The synergy coefficients are shown for the combination of MDA with vaccine of initial vaccine efficacy of 91% (upper row) and half-life of 0.61 years (left) or 1.5 years (right), and MDA with the lower vaccine efficacy of 50% (bottom row) and half-life of 0.61 years (left) or 1.5 years (right).

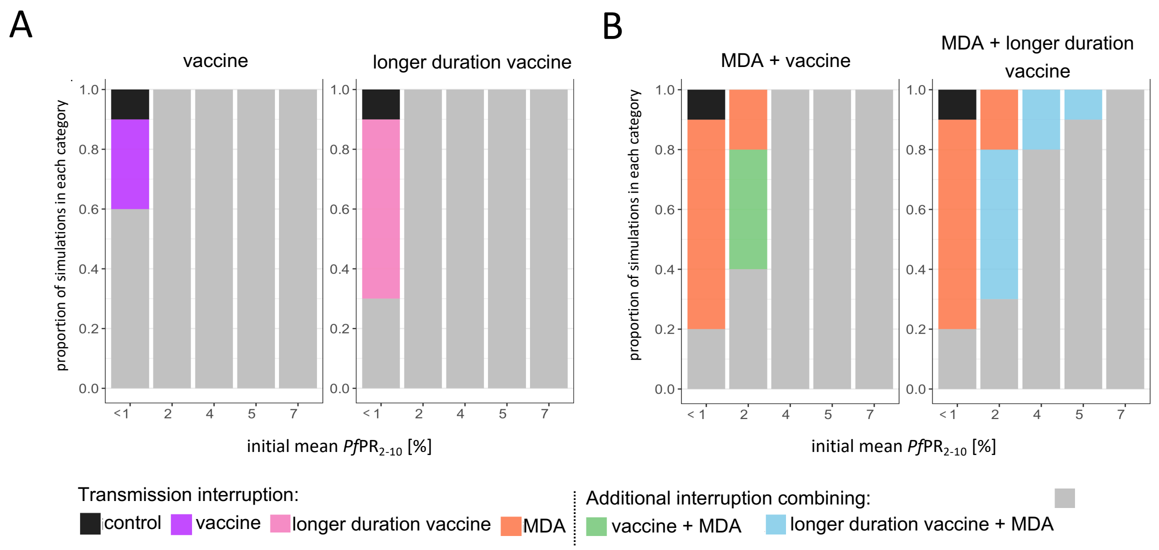

Figure S8: Interruption of transmission for mass vaccination and combined strategies with lower vaccine efficacy. Proportion of simulations in which interruption of transmission is estimated to be achieved with mass vaccination, **A,** or with mass vaccination combined to MDA, **B**, using a vaccine with initial efficacy of 50% and half-life of 0.61 years (left of each panel), and using a vaccine with initial efficacy of 50% and half-life of 1.5 years (right of each panel). Initial *Pf*PR$_{2\text{-}10}$ (%) levels are shown on the x-axis, and proportion of the simulations falling into each category are shown on the y-axis. All interventions were deployed for two years at a coverage of 60%. Categories of simulations are *i)* interruption of transmission occurred with no intervention at all, due to very low initial prevalence (black), *ii)* interruption of transmission occurred with single interventions, namely with mass vaccination with a lower efficacy vaccine with half-life of 0.61 years (purple) or longer duration vaccine (pink), or with MDA (orange), *iii)* interruption of transmission occurred only adding mass vaccination to MDA (green and blue using with a lower efficacy vaccine with half-life of 0.61 years or longer duration vaccine respectively), and *iv)* resurgence occurred and no interruption of transmission was achieved (grey).

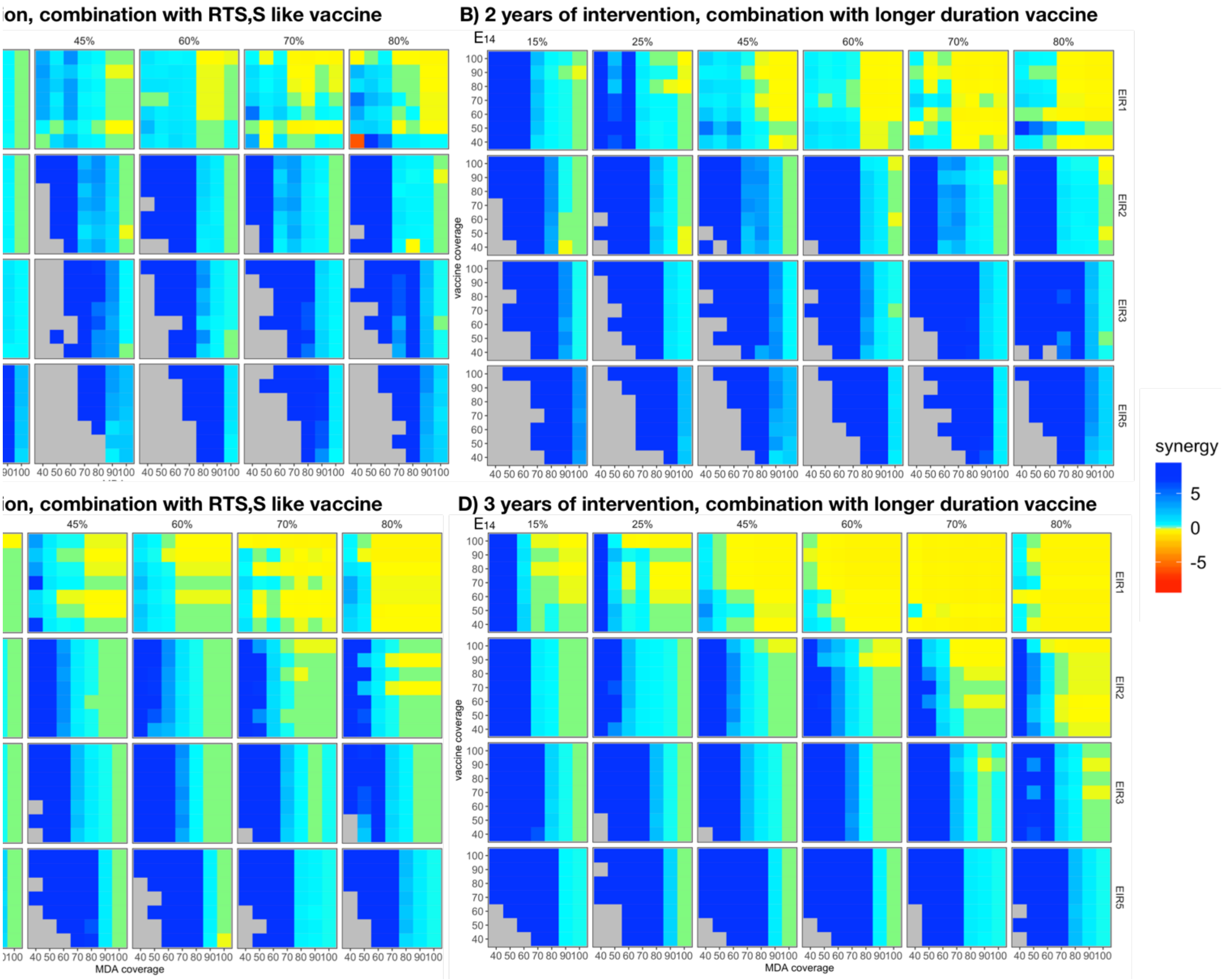

Figure S9: S
coefficient
vaccinatior
administrat
probability
transmissi
The x-axis i
coverage le
the y-axis in
coverage le
vaccination.
represents t
between the
strategies (s
Values of σ
blue, repres
that the esti
two combine
greater than
0, in light gr
combined in
more than a
less than 0,
imply less th
maximum le
by one or bo
intervention
represent se
resurgence
simulations,
can be calcu
represent th
years of interventions of MDA combined with mass vaccination with RTS,S like vaccine, **B)** represent the synergy c
:ions of MDA combined with mass vaccination with a vaccine with longer duration of protection, **C)** represent the syr
terventions of MDA combined with mass vaccination with RTS,S like vaccine and **D)** represent the synergy coefficie
IDA combined with mass vaccination with a vaccine with longer duration of protection. Results are shown for differe
it (14-day effective treatment probabilities) (rows) and different levels of EIR (columns).

ɪsurgence parameters for 2 years deployment of MDA, mass vaccination, or combination of both MDA and ɪported infections. The estimated parameters to individual regressions to each 10 simulations in each strategie ɪnce intervals for the estimated half-life, λ_{50}, representing the years after maximum prevalence was reached wher lence; the Hill's slope, representing the steepness of the logistic curve; and the 10% resurgence threshold, λ_{10}, re ce was reached where 10% of the resurgence occurred. The interventions are deployed during 2 years, initial pr ɔ and E_{14}=25%). In the combined strategies, estimates when vaccination and drugs are given to the same proport ɪified with [A], if not specified, a random coverage of the population is selected independently for each intervention. V and in the combined strategies specified by [B] initial vaccine efficacy against infection is lower at 50%.

	importation	half-life, λ_{50}		Hill slope		10% resurgence threshol	
		**EIR = 5; E_{14} = 25 % ; *Pf*PR_{2-10} = 9%**					
	0	3.68	[3.34 - 4.02]	3.55	[3.05 - 5.05]	2.00	[1.80 - 2.32
		3.68[A]	[3.21 – 3.86] [A]	3.78[A]	[2.61 – 4.48] [A]	1.92[*]	[1.43 - 2.31
sion		2.56[A,B]	[2.21 – 2.93] [A,B]	2.84 [A,B]	[2.02 – 3.21] [A,B]	1.20 [A,B]	[0.82 - 1.34
	2	3.29	[2.90 - 4.10]	3.54	[3.08 - 4.35]	1.83	[1.42 - 2.38
	20	2.36	[2.10 - 2.72]	2.42	[1.77 - 2.96]	0.94	[0.66 - 1.24
	0	4.00	[3.14-5.39]	3.87	[3.42- 5.97]	2.26	[1.71 -3.45]
sion	2	3.54	[2.84-4.29]	3.98	[3.09 -4.59]	2.09	[1.57-2.60]
	20	2.35	[1.97-2.99]	2.52	[1.94 -2.88]	0.98	[0.81-1.09]
	0	1.83	[1.46 - 2.52]	2.57	[1.93 - 3.06]	0.72	[0.60 - 1.19
	2	1.66	[1.41 - 1.95]	2.64	[1.97 - 3.02]	0.67	[0.56 - 0.86
	20	1.36	[1.20 - 1.54]	2.25	[1.84 - 2.62]	0.49	[0.41 - 0.58
	0	2.75	[2.11 - 3.59]	2.71	[1.52 - 3.59]	1.04	[0.64 - 1.68
	2	2.78	[2.09 - 3.32]	2.58	[1.78 - 3.34]	1.16	[0.92 - 1.51
ı	20	2.55	[2.01 - 3.19]	2.05	[1.35 - 3.12]	0.88	[0.56 - 1.07
	0	2.48	[2.26-2.76]	2.28	[1.81 -2.74]	0.92	[0.69 -1.18]
	2	2.54	[2.34-3.07]	2.39	[1.79-3.36]	0.98	[0.80 -1.30]
ı	20	1.98	[1.60-2.64]	2.12	[1.37-2.89]	0.71	[0.49-0.84]
		**EIR = 2; E_{14} = 45 % ; *Pf*PR_{2-10} = 4%**					
	0	6.78	[5.05 - 12.92]	3.12	[2.76 - 4.15]	3.46	[2.60 - 6.11
		7.25 [A]	[5.19 – 8.35] [A]	3.09 [A]	[1.67 - 4.22] [A]	3.44 [A]	[1.85 - 4.55
sion		6.37 [B]	[5.12 – 10.24] [B]	3.25 [B]	[2.87 – 5.46] [B]	6.37 [B]	[5.12 – 10.2
		6.31 [A.B]	[3.80 – 10.83] [A,B]	2.92 [A,B]	[1.49 - 4.73] [A,B]	2.98 [A,B]	[1.67 – 5.17

	2	6.43	[4.80 – 8.27]	2.94	[2.36 - 4.61]	3.02	[2.55 – 3.67
	20	3.12	[2.73 – 3.86]	2.36	[1.66 - 2.75]	1.16	[0.90 - 1.41
	0	9.74	[8.37 -62.41]	4.34	[3.14 -216.15]	5.80	[4.84 -61.25
**sion**	2	7.36	[5.33 -11.95]	2.76	[2.23-4.58]	3.93	[2.21-5.06]
	20	3.11	[2.71-3.25]	1.89	[1.62-3.30]	0.91	[0.81- 1.59]
	0	4.63	[3.21 – 9.57]	2.23	[1.71 – 4.38]	1.98	[1.30 - 3.19
	2	4.22	[3.62 – 6.00]	2.33	[150 – 2.94]	1.68	[0.91 - 1.86
	20	2.09	[1.72 – 2.60]	1.81	[1.33 – 2.71]	0.55	[0.41 – 0.88
	0	3.45	[2.09 – 6.09]	2.38	[1.25 – 4.76]	1.31	[0.56 – 2.30
	2	4.35	[2.95 – 5.52]	1.88	[1.00 – 4.83]	1.41	[0.48 – 2.05
**n**	20	3.60	[2.36 – 6.15]	2.52	[1.42 – 4.55]	1.58	[0.88 – 2.38
	0	4.01	[2.71- 6.93]	2.99	[1.37-4.03]	1.75	[1.24-2.22]
	2	4.99	[2.41-6.55]	1.55	[1.02-3.92]	1.09	[0.48-2.05]
**n**	20	3.12	[2.29-4.28]	2.56	[0.88-4.27]	1.24	[0.33-1.61]

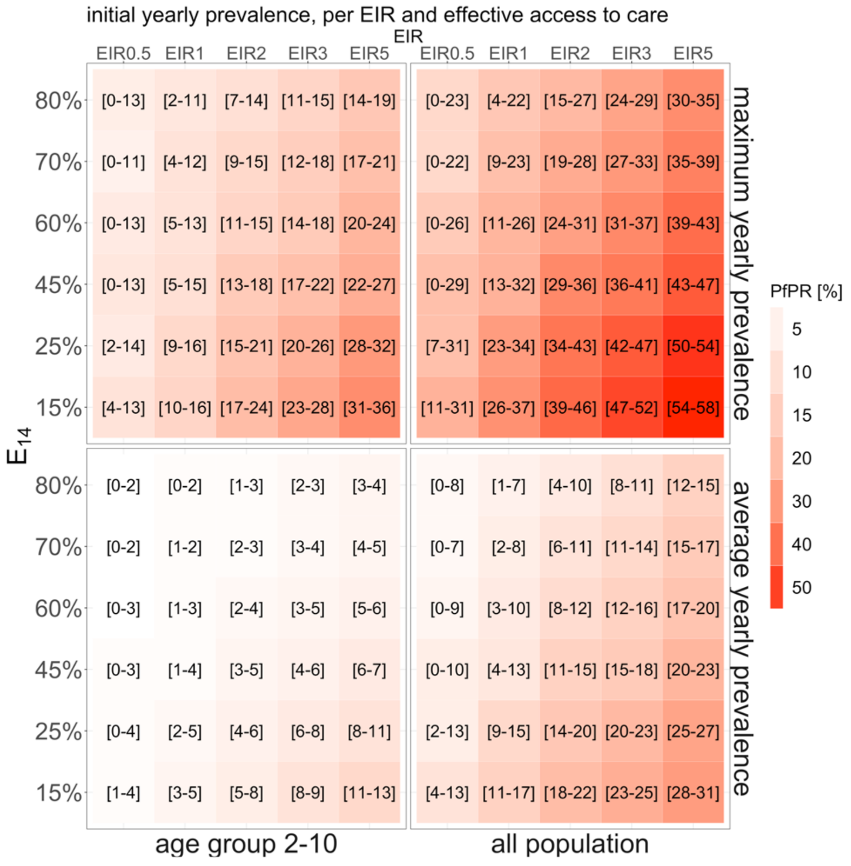

Figure S10: Relationship between entomological inoculation rate, EIR, and effective access to care, E_{14}, with *Pf*PR$_{2-10}$ and prevalence in all population. 5 different levels of EIR, from 0.5 to 5, are represented in the x-axis and 6 different levels of effective treatment E_{14}= 15%; 25%; 45%; 60%; 70% 80%. The corresponding prevalence with given access and EIR levels is shown by the color gradient, the upper plots representing the maximum prevalence in the seasonal setting and the lower plots the average yearly prevalence. The plots on the left show PfPR$_{2-10}$ levels and the plots on the right the prevalence in all population. Results are across 10 simulations for each setting, the median value across the simulations is indicated by the color gradient, and the 95% range is indicated in brackets

References

1. Smith T, et al. (2012) Ensemble modeling of the likely public health impact of a pre-erythrocytic malaria vaccine. *PLoS Med* 9(1). doi:10.1371/journal.pmed.1001157.
2. Crowell V, et al. (2012) Can we depend on case management to prevent re-establishment of P. falciparum malaria, after local interruption of transmission? *Epidemics* 4(1):1–8.
3. Brady OJ, et al. (2017) Role of mass drug administration in elimination of Plasmodium falciparum malaria: a consensus modelling study. *Lancet Glob Heal* 5(7):e680–e687.
4. Penny MA, et al. (2016) Public health impact and cost-effectiveness of the RTS,S/AS01 malaria vaccine: a systematic comparison of predictions from four mathematical models. *Lancet* 6736(15):1–9.
5. World Health Organization (2016) Weekly epidemiological record: relevé épidémiologique hebdomadaire. *Wkly Epidemiol Rec* (47):445–452.
6. Malaria Policy Advisory Committee Meeting (2015) Consensus modelling evidence to support the design of mass drug administration programmes (WHO, Geneva).
